# Glutamine-Functionalized mRNA Lipid Nanoparticles Block HIF-1α-Dependent Glycolytic Adaptation to Sensitize Azelaic Acid Therapy in Colorectal Cancer

**DOI:** 10.3390/molecules31213791

**Published:** 2026-10-22

**Authors:** Fei Teng, Yuanyuan Yang, An Xiong, Rongjian Zhou, Chencheng Shi, Chang Su, Xiaodong Shen, Mengxuan Yang, Ming Lei, Yiou Cao, Yi Chen, Puhua Wang, Shaoqun Liu

**Affiliations:** 1 Department of Gastrointestinal Surgery, Minhang Hospital, Fudan University, Shanghai 201199, China; prof_tengfei@fudan.edu.cn (F.T.); 21211360003@m.fudan.edu.cn (A.X.); 18111210075@fudan.edu.cn (R.Z.); shichencheng0510@163.com (C.S.); suchang_jx@126.com (C.S.); xds187@126.com (X.S.); yangmengxuan@fudan.edu.cn (M.Y.); leiming@fudan.edu.cn (M.L.); yioucao_doctor@126.com (Y.C.); 2 Department of General Surgery, People’s Hospital of Daguan County, Zhaotong 657400, China; 3 Department of Pathology, Minhang Hospital, Fudan University, Shanghai 201199, China; yuanyuanyang1982@outlook.com

**Keywords:** colorectal cancer, lipid nanoparticles, mRNA delivery, HIF-1α, metabolic plasticity, azelaic acid

## Abstract

Metabolic plasticity limits mitochondria-directed cancer therapy by enabling glycolytic compensation during respiratory stress. Our previous nano-energy interference (NEI) platform showed that coupling azelaic acid (AZA)-induced mitochondrial disruption with restriction of glucose utilization suppresses colorectal cancer (CRC), but the upstream program coordinating this adaptive response remained unresolved. Here, we identify HIF-1α-dependent glycolytic adaptation as a key response to AZA-induced respiratory inhibition and develop glutamine-functionalized lipid nanoparticles (G-LNPs) to deliver dominant-negative HIF-1α (dnHIF-1α) mRNA. G-LNPs were approximately 105 nm in diameter, achieved >90% mRNA encapsulation, and enhanced uptake and functional reporter mRNA expression in ASCT2-expressing MC38 cells in a GPNA-sensitive manner. AZA suppressed mitochondrial respiration while activating HIF-responsive transcription, glycolytic activity, lactate production, and GLUT1, HK2, LDHA, and PDK1 expressions. G-LNP-delivered dnHIF-1α blocked this compensatory response without materially intensifying respiratory inhibition, thereby deepening ATP depletion and producing a distinct treatment-associated metabolomic state. Following treatment withdrawal and equal viable cell replating, the combination caused persistent impairment of regrowth and clonogenic capacity. In subcutaneous MC38 tumors, combined treatment produced the greatest tumor growth inhibition without overt systemic toxicity. Together, these findings support transient disruption of HIF-dependent metabolic adaptation as a strategy to convert partially recoverable mitochondrial stress into a durable loss of CRC cell fitness.

## 1. Introduction

Colorectal cancer (CRC) is the third most frequently diagnosed malignancy and the second leading cause of cancer-related death worldwide, with more than 1.9 million new cases and approximately 900,000 deaths reported in 2022 [1]. Despite advances in screening, surgery, targeted therapy and immunotherapy, durable disease control remains difficult in advanced and treatment-refractory CRC. In addition to genomic heterogeneity, therapeutic resistance is reinforced by metabolic plasticity, which enables tumor cells to redistribute nutrient utilization and ATP production in response to oxygen limitation, nutrient deprivation and treatment-induced stress [2,3,4]. Consequently, inhibition of a single energy-producing pathway may be buffered by compensatory engagement of an alternative metabolic program. Therapeutic strategies that impose bioenergetic stress while simultaneously preventing adaptation to that stress may therefore produce more sustained disruption of tumor cell metabolism.

Although aerobic glycolysis is a prominent feature of CRC, tumor cells retain functional mitochondria and can dynamically redistribute energy production between oxidative phosphorylation and glycolysis [2,3,5]. Hypoxia-inducible factor 1 (HIF-1) is a central regulator of this metabolic flexibility. Following stabilization, HIF-1α dimerizes with HIF-1β and activates transcriptional programs that increase glucose uptake and glycolytic flux, including induction of SLC2A1/GLUT1, HK2 and LDHA [6]. HIF-1 also induces pyruvate dehydrogenase kinase 1 (PDK1), thereby restricting pyruvate entry into mitochondrial oxidation [7], and can reduce mitochondrial oxygen consumption under hypoxic stress [8]. Collectively, these responses facilitate a shift toward glycolytic ATP production when mitochondrial oxidative metabolism becomes constrained. If this compensatory capacity is insufficient, energetic stress can activate AMPK-dependent metabolic checkpoints and constrain mTORC1-linked anabolic activity [9], providing a mechanistic connection between failed energy adaptation and loss of proliferative fitness. HIF-1α therefore represents a plausible upstream regulator of the adaptive response to mitochondria-directed metabolic stress in CRC cells.

Azelaic acid (AZA), a nine-carbon dicarboxylic acid, served as the mitochondrial-disrupting component of our previously reported nano-energy interference (NEI) strategy. In that study, AZA and Fe_3_O_4_ were incorporated into 2-deoxy-D-glucose (2-DG)-decorated lipid nanoparticles to generate the MRI-visible DAF@LNP platform [10]. In HCT116 CRC models, the AZA-containing formulation disrupted mitochondrial structure and respiration, increased reactive oxygen species and enhanced glucose demand. Concurrent restriction of glucose utilization by 2-DG intensified ATP depletion and suppressed tumor growth and experimental liver metastasis [10]. These findings established the central NEI concept that mitochondria-directed stress can be potentiated by simultaneously restricting the metabolic pathway recruited to compensate for that stress.

Building on this established NEI framework, the present study retained AZA as an experimentally characterized respiratory/metabolic stress condition to investigate whether blocking the adaptive metabolic response to AZA-associated respiratory dysfunction could enhance its antitumor activity. AZA was not selected as an established clinical metabolic inhibitor for cancer therapy; rather, its inclusion ensured continuity with the previously developed NEI platform, in which AZA-associated mitochondrial and respiratory phenotypes and their compatibility with the lipid nanoparticle formulation had already been demonstrated in CRC models [10]. This selection also enabled a mechanistically focused investigation. Whereas 2-DG acts downstream by restricting glycolytic substrate utilization, metformin, phenformin, and other complex I-targeting agents have distinct pharmacological properties and systemic exposure considerations, and would not be expected to interrogate precisely the same adaptive response to AZA-associated respiratory stress. Accordingly, AZA was employed as an experimentally characterized respiratory/metabolic stress condition, without implying superiority over clinically investigated metabolic inhibitors. Further comparative dose–response studies using matched mitochondrial and glycolytic inhibitors will be required to determine whether the observed adaptation blockade effect can be generalized across different primary metabolic stresses.

The previous platform, however, intervened primarily at the level of glucose utilization and did not directly interrogate the upstream transcriptional program coordinating glycolytic adaptation. In this first-generation NEI design, 2-DG restricted compensatory glucose utilization downstream of the adaptive response. Respiratory stress, by contrast, can engage a broader HIF-dependent transcriptional program that coordinately regulates glucose transport, glycolytic enzymes, lactate production and mitochondrial substrate utilization. We therefore reasoned that targeting HIF-1α could extend NEI from downstream nutrient restriction to programmable upstream control of the adaptive program itself. Such an approach could simultaneously suppress multiple components of glycolytic compensation, rather than inhibiting a single metabolic reaction and, in principle, convert a partially recoverable mitochondrial stress into a more persistent loss of metabolic fitness. This transition from blocking a compensatory substrate pathway to disabling the regulatory program that organizes metabolic escape represents a mechanistic evolution of the NEI concept.

A dominant-negative HIF-1α (dnHIF-1α) provides a genetic strategy for transient interference with HIF-dependent transcription. Removal of the HIF-1α transactivation domain generates a protein capable of interfering with productive HIF transcriptional activity without activating downstream HIF-responsive genes. A HIF-1α construct lacking the transactivation domain has previously been shown to suppress HIF-responsive transcription and restore drug sensitivity in hypoxic tumor cells [11]. Delivery of such a dominant-negative regulator as messenger RNA (mRNA) is particularly attractive because mRNA enables rapid, transient and non-integrating protein expression [12,13,14]. Naked mRNA, however, is susceptible to extracellular degradation and exhibits inefficient cellular entry. Lipid nanoparticles (LNPs) can protect mRNA cargo, facilitate cellular uptake and endosomal escape, and support intracellular protein expression [12,13,14]. Efficient delivery to tumor cells nevertheless remains a major challenge for systemic mRNA-LNP therapy.

At the payload level, the present strategy differs from the small-molecule inhibition and nucleic acid-silencing approaches commonly used to interfere with HIF signaling in nanomedicine studies. A dominant-negative HIF-1α mRNA supplies a transient, non-integrating intracellular competitor that can inhibit HIF-dependent transcription without requiring genomic modification. At the carrier level, the current G-LNP is also distinct from the earlier DAF@LNP. DAF@LNP was a compact, multifunctional theranostic formulation that co-loaded Fe_3_O_4_ and AZA and displayed 2-DG to combine MRI monitoring, mitochondrial stress, and downstream glucose uptake restriction [10]. By contrast, G-LNP uses an ionizable mRNA-LNP core containing SM-102, DSPC, cholesterol, Gln-PEG-DMG, and mPEG-DMG, with glutamine displayed at low molar fraction and dnHIF-1α mRNA as the functional cargo. The carrier is therefore optimized for protection, cytosolic release, and transient protein expression, rather than for inorganic imaging or direct substrate competition. Conventional ionizable LNPs can support mRNA expression, but generally lack a cell phenotype-associated ligand [13]; SORT formulations primarily alter organ-level distribution; and receptor-targeted carriers remain sensitive to target heterogeneity, ligand accessibility, and protein corona remodeling [15,16]. The originality of the present study lies in combining a nutrient-associated cell interaction strategy with transient dominant-negative HIF-1α mRNA delivery to extend NEI from downstream metabolic co-interference toward programmable control of the upstream adaptation program. We do not interpret this design as universally superior to DAF@LNP or other mRNA platforms; rather, it addresses a different mechanistic and delivery problem.

The neutral amino acid transporter ASCT2 (SLC1A5) provides a metabolically relevant cell surface feature that may be exploited to enhance the interaction of mRNA nanoparticles with CRC cells. ASCT2 contributes to glutamine uptake and thereby supports carbon and nitrogen metabolism, as well as nutrient-sensitive growth signaling. In CRC, oncogenic KRAS has been associated with increased ASCT2 expression, whereas high ASCT2 abundance has been associated with greater tumor depth and vascular invasion in KRAS-mutant tumors [17]. KRAS-driven YAP1 signaling can additionally increase SLC1A5 and other amino acid transporters, promoting amino acid acquisition by CRC cells [18]. On this basis, we reasoned that glutamine functionalization of LNPs could enhance nanoparticle–cell interactions in ASCT2-expressing CRC cells and thereby increase productive mRNA delivery. Importantly, this design was intended to exploit a tumor-associated nutrient acquisition phenotype to enhance delivery, rather than to presume ASCT2-specific nanoparticle internalization, thereby linking a delivery-relevant metabolic phenotype to inhibition of a distinct HIF-dependent adaptation pathway.

Glutamine was selected as the targeting moiety because it is an endogenous, chemically compact nutrient ligand that can be displayed at low molar fraction without the steric burden associated with antibody- or protein-based ligands. Its potential advantage is biological, rather than merely structural: ASCT2 expression can be associated with a nutrient acquisition phenotype that is linked to glutamine utilization in subsets of CRC, creating a route to couple delivery to a metabolic state that is mechanistically related to the therapeutic objective. A previous polymeric carrier study demonstrated multivalent interaction of glutamine-functionalized polymers with ASCT2-overexpressing tumors, but that system did not address ionizable LNP-mediated mRNA delivery [19]. Glutamine is not expected to provide the same absolute specificity as a high-affinity antibody or peptide ligand; free glutamine, transporter redundancy, and protein corona formation may attenuate selectivity. We therefore interpret G-LNP as a metabolically associated targeting strategy, not as a receptor-exclusive system.

**Scheme 1:**
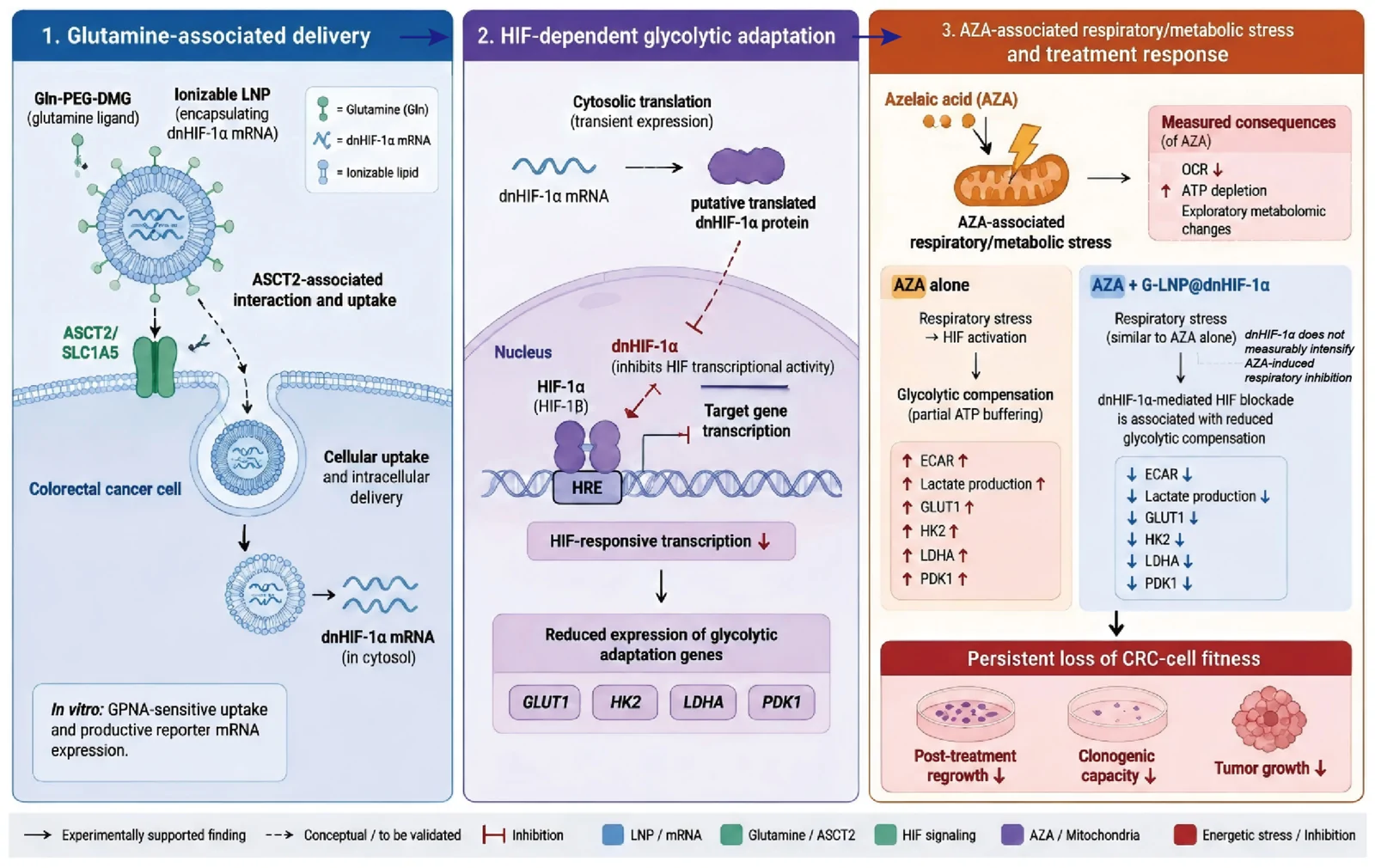
Mechanistic overview of glutamine-associated dnHIF-1α mRNA delivery and AZA response in colorectal cancer. (1) Gln-PEG-DMG-functionalized ionizable lipid nanoparticles encapsulating dnHIF-1α mRNA interact with ASCT2/SLC1A5-expressing colorectal cancer cells and support cellular uptake and intracellular mRNA delivery. Under the tested in vitro conditions, this process was glutamine-sensitive and GPNA-sensitive, and was associated with enhanced productive reporter mRNA expression. (2) Cytosolic delivery and transient translation of dnHIF-1α produce a dominant-negative protein that interferes with HIF-1α/HIF-1β-dependent transcription at hypoxia-response elements, thereby reducing HIF-responsive transcription and the expression levels of glycolytic adaptation genes, including GLUT1, HK2, LDHA, and PDK1. (3) Azelaic acid (AZA) is used here as an experimental respiratory/metabolic stress, a condition associated with reduced oxygen consumption rate and ATP depletion. AZA alone activates HIF-dependent glycolytic compensation, reflected by increased extracellular acidification rate, lactate production, and glycolytic gene expression, thereby partially buffering ATP loss. In the combined condition, G-LNP@dnHIF-1α suppresses this adaptive response without materially increasing the primary AZA-induced respiratory defect, resulting in reduced glycolytic activity, deeper ATP depletion, a distinct treatment-associated metabolomic state, impaired post-treatment regrowth and clonogenic capacity, and enhanced tumor growth inhibition. Red upward arrows (↑) in the AZA-alone panel indicate increased ECAR, lactate production, and GLUT1, HK2, LDHA, and PDK1 expression relative to the corresponding control. Blue downward arrows (↓) in the combined-treatment panel indicate reductions in these readouts relative to AZA treatment without dnHIF-1α. Solid arrows indicate experimentally supported findings, whereas dashed arrows indicate conceptual relationships or mechanisms requiring further validation. Inhibition symbols denote negative regulatory effects.

Here, we developed glutamine-functionalized LNPs carrying dnHIF-1α mRNA (G-LNP@dnHIF-1α mRNA) and combined them with AZA to test whether transient blockade of HIF-dependent adaptation could convert mitochondrial respiratory stress into sustained metabolic and proliferative impairment (Scheme 1). We hypothesized that AZA-induced respiratory inhibition would engage HIF-dependent glycolytic compensation, whereas G-LNP-mediated dnHIF-1α expression would prevent this adaptive response without materially increasing the primary mitochondrial defect. Accordingly, the central premise of this study was that therapeutic vulnerability could be created not by simply intensifying mitochondrial injury, but by preventing CRC cells from metabolically adapting to that injury. We therefore examined ASCT2-associated nanoparticle–cell interaction and productive mRNA expression, HIF transcriptional activity, oxidative and glycolytic metabolism, ATP and lactate production, untargeted metabolomic state differences under AZA stress, and post-treatment proliferative fitness. Therapeutic activity and preliminary systemic tolerability were subsequently evaluated in subcutaneous MC38 tumors. This study thereby advances NEI from downstream restriction of glucose utilization toward programmable inhibition of an upstream adaptive regulator engaged by mitochondrial stress.

## 2. Results and Discussion

### 2.1. Construction and Physicochemical Characterization of G-LNP@dnHIF-1α mRNA

**Figure 1:**
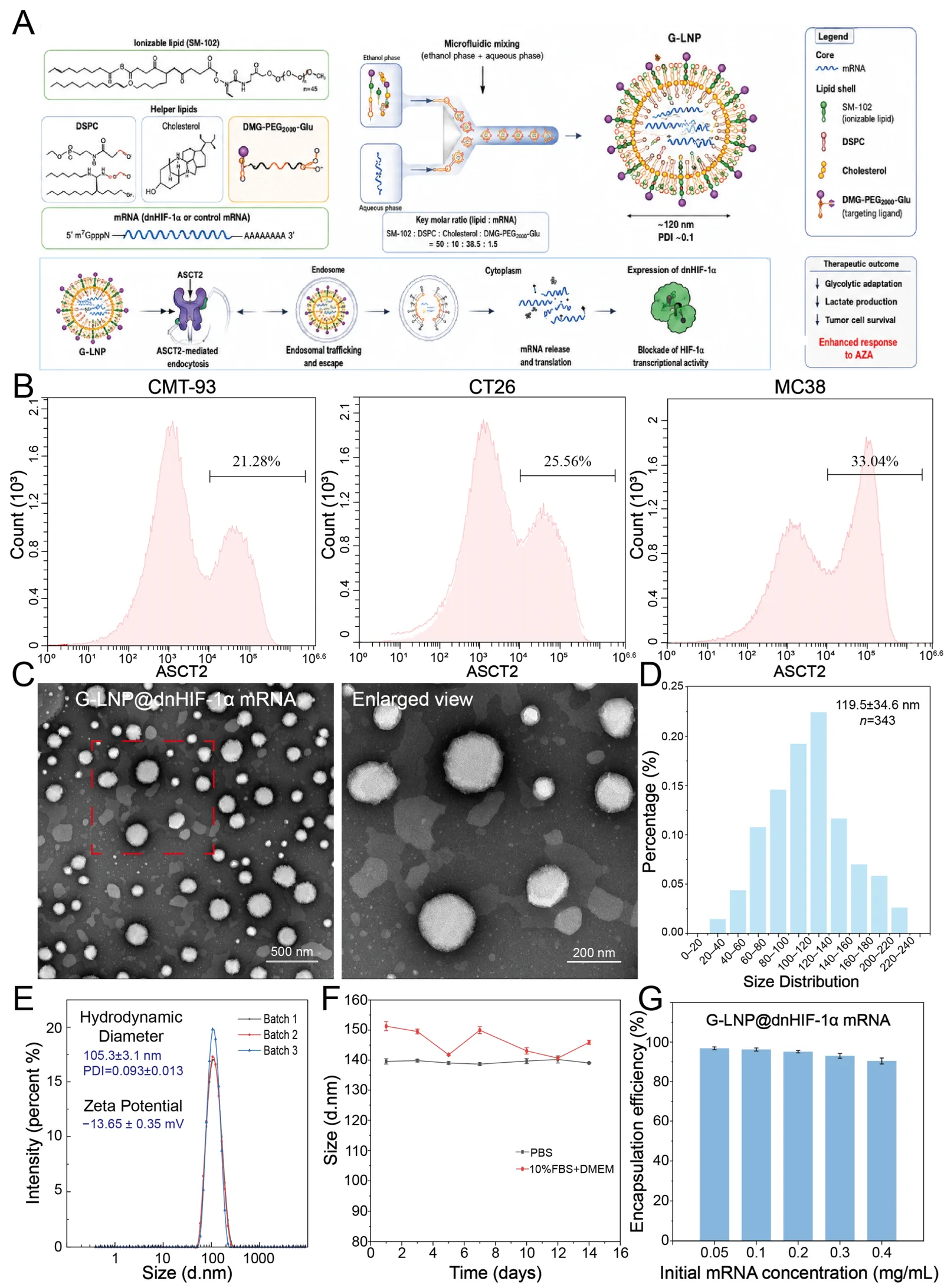
Construction and physicochemical characterization of glutamine-functionalized G-LNP@dnHIF-1α mRNA. (**A**) Schematic of Gln-PEG-DMG synthesis, microfluidic nanoparticle assembly, and the proposed delivery concept. DMG-PEG2000-NH2 was coupled to N-Boc-L-Glu-OαBn using DMT-MM and DIPEA in DMF, followed by deprotection to generate Gln-PEG-DMG. The lipid phase contained SM-102, DSPC, cholesterol, Gln-PEG-DMG, and mPEG-DMG at a molar ratio of 50:10:38.5:0.3:1.2, and the aqueous phase contained dnHIF-1α mRNA in 25 mM sodium acetate (pH 4.0). The two phases were mixed microfluidically at a flow rate ratio of 3:1, a total flow rate of 12 mL min^−1^, and an N/P ratio of 6, followed by dilution, ultrafiltration, buffer exchange into PBS, and addition of 8% sucrose. Surface-displayed glutamine was designed to enhance interaction with ASCT2-expressing CRC cells, whereas the ionizable lipid core enabled mRNA encapsulation and intracellular delivery. (**B**) Representative flow cytometric histograms of cell surface ASCT2 expression in CMT-93, CT26, and MC38 cells. The gated ASCT2-positive fractions shown were 21.28%, 25.56%, and 33.04%, respectively. Representative data from one of three independent experiments are shown. (**C**) Representative TEM images of G-LNP@dnHIF-1α mRNA and an enlarged view of the indicated region. Scale bars, 500 nm (**left**) and 200 nm (**right**). (**D**) TEM-derived particle size distribution based on 343 particles; mean diameter, 119.5 ± 34.6 nm. (**E**) Intensity-weighted hydrodynamic size distribution measured by DLS. The mean hydrodynamic diameter was 105.3 ± 3.1 nm, the polydispersity index was 0.093 ± 0.013, and the zeta potential was −13.65 ± 0.35 mV (*n* = 3 independent measurements). (**F**) Hydrodynamic diameter of G-LNP@dnHIF-1α mRNA monitored for 14 days in PBS or DMEM containing 10% FBS (*n* = 3). (**G**) mRNA encapsulation efficiency at initial mRNA concentrations of 0.05, 0.1, 0.2, 0.3, and 0.4 mg mL^−1^ (*n* = 3). DLS, dynamic light scattering; DMEM, Dulbecco’s modified Eagle medium; FBS, fetal bovine serum; PDI, polydispersity index; TEM, transmission electron microscopy.

To establish an mRNA delivery platform for subsequent metabolic intervention studies, Gln-PEG-DMG was custom-synthesized and incorporated into ionizable lipid nanoparticles carrying dnHIF-1α mRNA. Figure 1A illustrates the chemical structure and synthetic route of the custom-synthesized Gln-PEG-DMG, together with the subsequent microfluidic formulation of G-LNP@dnHIF-1α mRNA. The resulting lipid was formulated with SM-102, DSPC, cholesterol, Gln-PEG-DMG, and mPEG-DMG at a molar ratio of 50:10:38.5:0.3:1.2 and microfluidically mixed with dnHIF-1α mRNA at an N/P ratio of 6. Following dilution, ultrafiltration, buffer exchange into PBS, and addition of 8% sucrose, the resulting formulation was designated G-LNP@dnHIF-1α mRNA. In this design, surface-displayed glutamine was introduced to enhance interactions with ASCT2-expressing CRC cells, whereas the ionizable lipid nanoparticle core provided protection and intracellular delivery of the mRNA cargo.

Because the rationale for glutamine functionalization depends on the availability of ASCT2 at the tumor cell surface, we first examined ASCT2 expression in three murine CRC cell lines. Flow cytometric analysis identified ASCT2-positive fractions of 21.28% in CMT-93 cells, 25.56% in CT26 cells, and 33.04% in MC38 cells (Figure 1B and Supplementary Figure S1), demonstrating detectable but heterogeneous surface expression across the candidate models. MC38 cells were selected for subsequent experiments because they showed the highest ASCT2-associated signal among the tested CRC cell lines.

Transmission electron microscopy (TEM) revealed predominantly spherical nanoparticles with a mean diameter of 119.5 ± 34.6 nm based on analysis of 343 particles (Figure 1C,D). Dynamic light scattering (DLS) yielded an intensity-weighted hydrodynamic diameter of 105.3 ± 3.1 nm, with a polydispersity index of 0.093 ± 0.013 and a zeta potential of −13.65 ± 0.35 mV (Figure 1E). The comparable particle dimensions obtained by TEM and DLS were consistent with a well-defined nanoscale formulation. During 14 days of monitoring, the mean hydrodynamic diameter showed no progressive increase in PBS and only limited variation in complete culture medium, indicating the absence of substantial time-dependent aggregation under the tested conditions (Figure 1F).

Encapsulation efficiency remained at or above approximately 90% across initial mRNA concentrations of 0.05–0.4 mg mL^−1^ (Figure 1G), indicating consistently high mRNA loading over the tested input range. We next used GFP mRNA as a reporter to determine whether the formulation supported functional protein expression. At 24 h after transfection, G-LNP@GFP mRNA achieved a higher GFP-positive fraction than either non-targeted LNP@GFP mRNA or Lipofectamine 3000@GFP mRNA (Supplementary Figure S2). Together, these results establish that G-LNPs possess well-defined nanoscale characteristics, high mRNA encapsulation efficiency, colloidal stability under the tested conditions, and the capacity to support functional reporter expression, providing the formulation basis for subsequent evaluation of cellular uptake and dnHIF-1α delivery.

These physicochemical parameters should be interpreted as enabling conditions, rather than as mechanistic evidence by themselves. The approximately 120 nm hydrodynamic size and low PDI indicate a relatively uniform dispersion, while the mildly negative zeta potential may reduce nonspecific electrostatic interactions, but can also limit electrostatic cell association. The low Gln-PEG-DMG fraction may help preserve colloidal stability while retaining ligand accessibility, and high encapsulation with short-term size stability supports reproducible mRNA dosing. However, DLS and zeta-potential measurements do not report protein corona composition, endosomal escape, cytosolic release, or translation efficiency. GFP expression integrates particle association, intracellular trafficking, RNA release, and translation; it should therefore not be interpreted as a direct readout of any single material parameter. The matched non-targeted LNP comparison supports a functional contribution of glutamine under the tested conditions, but does not establish that size, surface charge, or ligand density is individually causal. Future structure performance studies should vary glutamine density while matching core composition, particle size, PDI, and PEG content.

### 2.2. Glutamine Functionalization Enhances ASCT2-Associated Uptake and Productive mRNA Delivery in MC38 Cells

**Figure 2:**
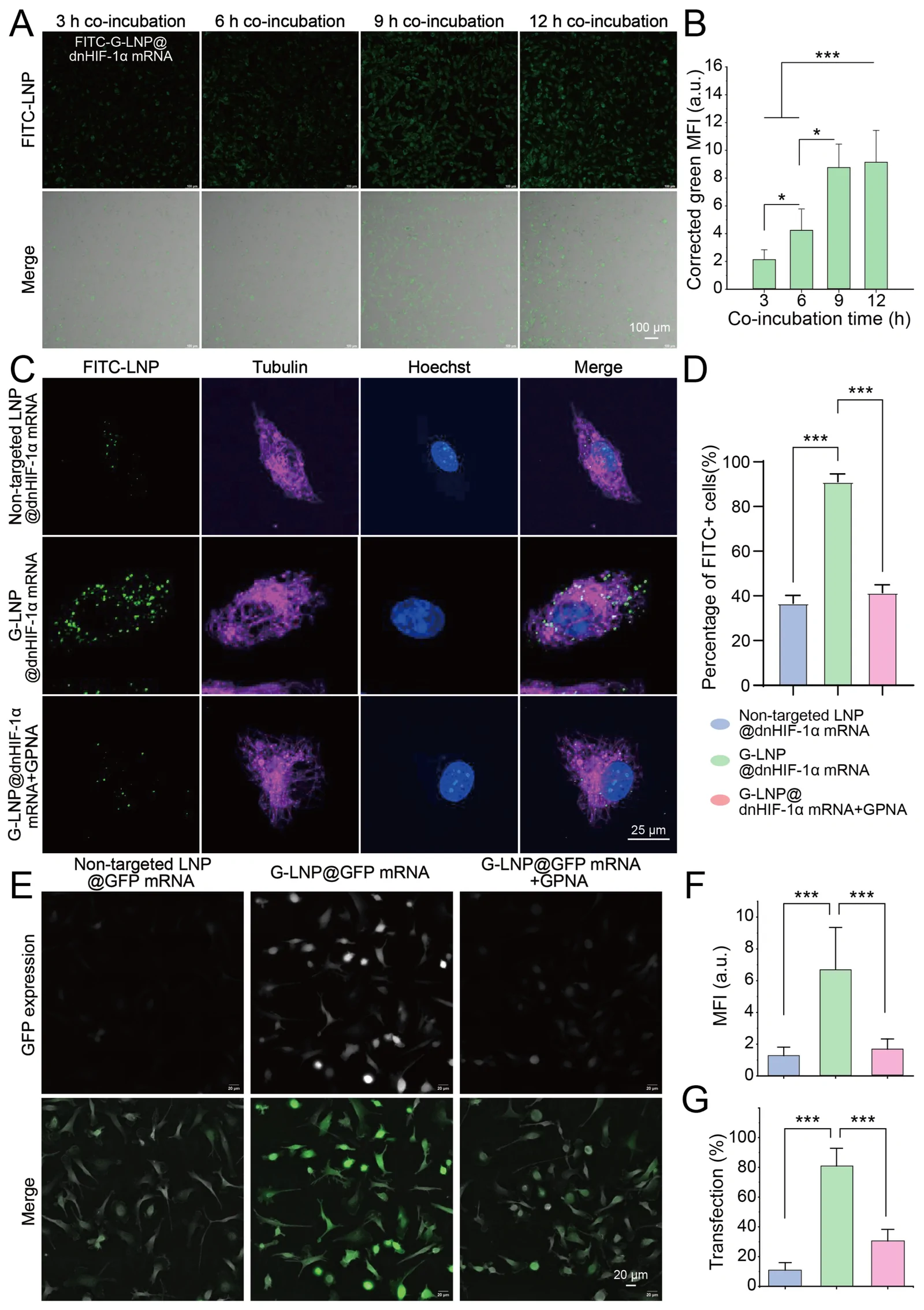
Glutamine functionalization enhances ASCT2-associated cellular uptake and productive mRNA delivery in MC38 cells. (**A**) Representative fluorescence micrographs of MC38 cells incubated for 3, 6, 9, or 12 h with FITC-labeled G-LNP@dnHIF-1α mRNA. The upper row shows the FITC channel, and the lower row shows merged fluorescence and transmitted light images. Scale bar, 100 μm. (**B**) Background-corrected green mean fluorescence intensity (MFI) measured at the indicated incubation times (*n* = 3 independent experiments). (**C**) Representative high-magnification fluorescence images of MC38 cells after 12 h of incubation with non-targeted LNP@dnHIF-1α mRNA, G-LNP@dnHIF-1α mRNA, or G-LNP@dnHIF-1α mRNA in the presence of GPNA. Green, FITC-labeled LNPs; magenta, α-tubulin; blue, Hoechst-stained nuclei. Scale bar, 25 μm. (**D**) Percentage of FITC-positive cells determined from the images represented in (**C**). (**E**) Representative GFP-channel and merged fluorescence images of MC38 cells treated with non-targeted LNP@GFP mRNA, G-LNP@GFP mRNA, or G-LNP@GFP mRNA in the presence of GPNA and imaged 12 h after nanoparticle addition. Scale bar, 20 μm. (**F**) Semi-quantitative GFP MFI derived from the images represented in (**E**) (*n* = 3 independent experiments). (**G**) Percentage of GFP-positive cells determined by image analysis. For all nanoparticle treatments, LNPs were applied at an mRNA-equivalent concentration of 0.5 μg mL^−1^. Where indicated, cells were pretreated with 1 mM GPNA for 30 min, and GPNA was maintained throughout nanoparticle exposure. Quantitative data are presented as mean ± SD. Statistical significance was assessed by one-way ANOVA followed by Tukey’s multiple comparisons test; comparisons correspond to the brackets shown. * *p* < 0.05 and *** *p* < 0.001; ns, not significant. FITC, fluorescein isothiocyanate; GPNA, L-γ-glutamyl-p-nitroanilide; MFI, mean fluorescence intensity.

Having established the physicochemical properties and expression competence of the formulation, we next examined whether glutamine functionalization enhanced cellular association and mRNA delivery in ASCT2-expressing CRC cells. MC38 cells were selected because they exhibited the highest ASCT2-positive fraction among the three murine CRC cell lines screened in Figure 1B. FITC-conjugated cholesterol was incorporated into the lipid phase to enable fluorescence-based tracking of the nanoparticles. Following incubation with FITC-labeled G-LNP@dnHIF-1α mRNA, cell-associated fluorescence increased progressively from 3 to 12 h, with background-corrected mean fluorescence intensity (MFI) rising markedly between 3 and 9 h and changing only modestly thereafter (Figure 2A,B). These kinetics indicated that cellular association approached a plateau by 9–12 h and supported the use of a 12 h incubation for subsequent comparative analyses.

High-magnification fluorescence imaging at 12 h showed relatively sparse FITC-positive puncta in cells exposed to non-targeted LNP@dnHIF-1α mRNA, whereas G-LNP@dnHIF-1α mRNA produced abundant punctate fluorescence throughout the tubulin-demarcated cytoplasm, including the perinuclear region (Figure 2C). Quantitative image analysis showed that the FITC-positive cell fraction increased from approximately 36% with non-targeted LNPs to approximately 90% with G-LNPs (Figure 2D). Addition of L-γ-glutamyl-p-nitroanilide (GPNA), an inhibitor of neutral amino acid transport, reduced the FITC-positive fraction to approximately 40%, approaching the level observed with non-targeted LNPs. The marked attenuation of the G-LNP uptake advantage in the presence of GPNA supports a glutamine-sensitive and ASCT2-associated contribution to enhanced nanoparticle–cell interaction.

We next asked whether this increase in cellular association translated into productive mRNA expression. MC38 cells were treated with non-targeted LNP@GFP mRNA, G-LNP@GFP mRNA, or G-LNP@GFP mRNA in the presence of GPNA and analyzed 12 h after nanoparticle addition. G-LNP@GFP mRNA produced a substantially stronger and more widespread GFP signal than the non-targeted formulation, whereas GPNA markedly attenuated reporter expression (Figure 2E and Supplementary Figure S2). Semi-quantitative image analysis yielded a GFP MFI of approximately 6.6 arbitrary units for G-LNPs, compared with 1.3 for non-targeted LNPs and 1.6 after GPNA competition (Figure 2F). Consistently, the estimated GFP-positive fraction increased from approximately 10% with non-targeted LNPs to approximately 80% with G-LNPs, and decreased to approximately 30% in the presence of GPNA (Figure 2G).

The concordance between fluorescent lipid association and GFP expression indicates that glutamine functionalization enhanced not only nanoparticle association with MC38 cells, but also productive delivery of mRNA to a translationally competent intracellular compartment. Together with the GPNA competition results, these findings identify Gln-PEG-DMG as a functional ligand component that enhances ASCT2-associated cellular uptake and reporter mRNA expression, providing the delivery basis for subsequent experiments with dnHIF-1α mRNA.

Because GPNA inhibits more than one neutral amino acid transport process [20], the competition experiment cannot by itself assign causality specifically to ASCT2. The present data therefore support glutamine-sensitive, ASCT2-associated nanoparticle interaction and productive mRNA expression, but do not establish ASCT2-specific receptor-mediated internalization. Definitive validation will require SLC1A5 loss-and-rescue experiments, competition with free L-glutamine or a validated ASCT2-selective inhibitor, comparison across isogenic low- and high-ASCT2 cells, ligand density titration, and direct measurements that distinguish surface binding, internalization, endosomal escape, and cytosolic mRNA release.

### 2.3. HIF-Dependent Glycolytic Compensation Buffers ATP Loss During AZA-Associated Respiratory Stress

**Figure 3:**
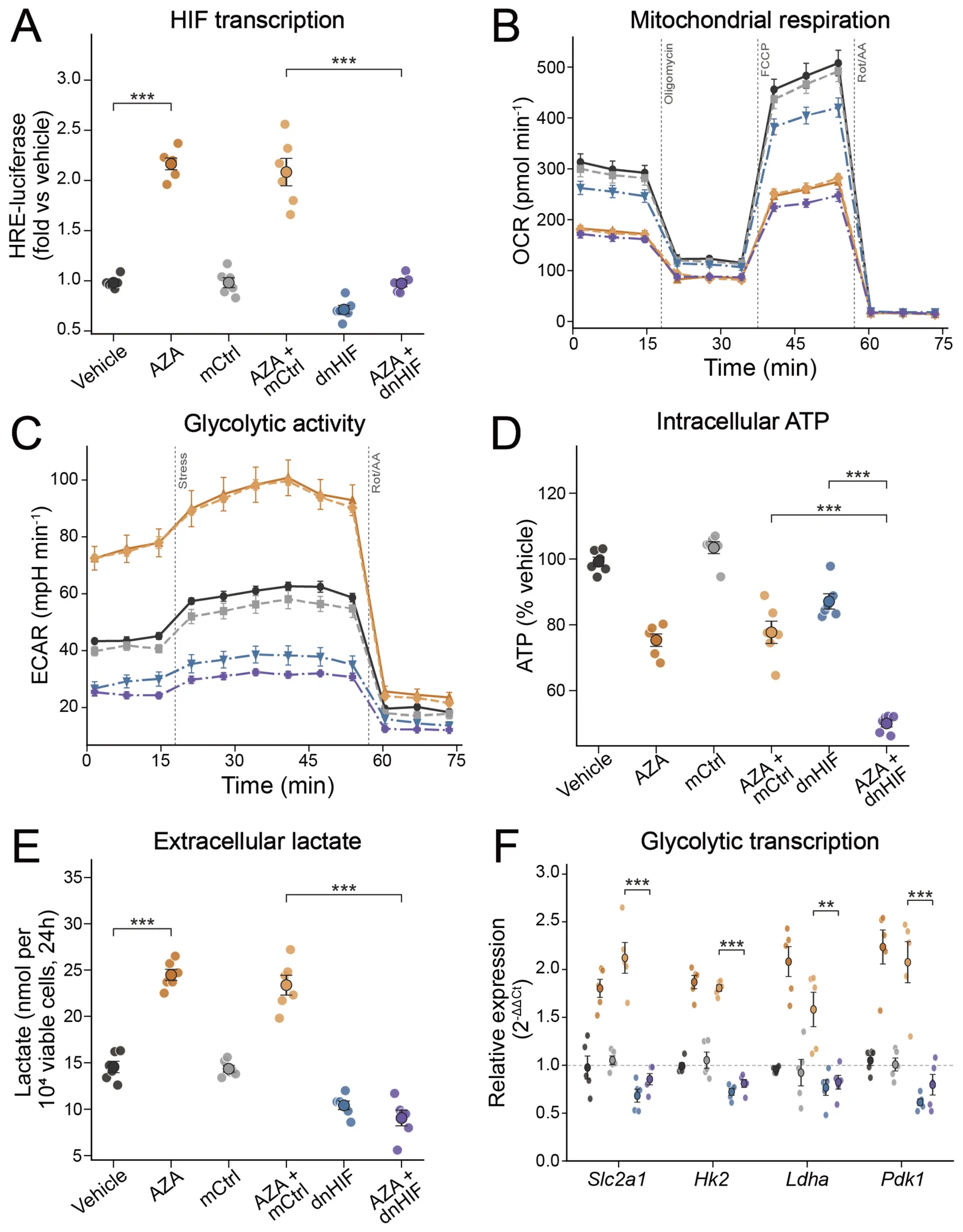
HIF-dependent glycolytic compensation buffers ATP loss during AZA-associated respiratory stress. For groups receiving mRNA-LNPs, cells were pre-exposed to the indicated formulation at an mRNA-equivalent concentration of 0.5 μg mL^−1^ for 12 h. Where indicated, AZA was then added at the nominal in vitro concentration of 5 mM, and cells were incubated for an additional 24 h; this exposure was used as an experimental respiratory/metabolic stress condition and is not intended to represent a plasma-equivalent exposure to the in vivo regimen. Potential ionic or osmotic contributions to the nominal AZA exposure were not separately tested. (**A**) HIF-responsive element (HRE)-luciferase activity in the indicated treatment groups. (**B**) Oxygen consumption rate (OCR) during mitochondrial stress testing. Dashed vertical lines indicate sequential addition of oligomycin, FCCP, and rotenone/antimycin A. (**C**) Extracellular acidification rate (ECAR) during the corresponding metabolic stress assay, used as a readout of glycolytic activity. Dashed vertical lines indicate stress induction and rotenone/antimycin A addition. (**D**) Intracellular ATP content normalized to the vehicle group. (**E**) Extracellular lactate accumulated over 24 h and normalized to viable cell number. (**F**) Relative expression levels of *Slc2a1*, *Hk2*, *Ldha*, and *Pdk1* determined by quantitative PCR and calculated using the 2^−ΔΔCt^ method relative to vehicle. Points represent independent biological replicates; central markers and lines indicate means, and error bars indicate s.e.m. *n* = 6 independent biological replicates for (**A**–**E**) and *n* = 5 for (**F**). Group comparisons of HRE activity, basal OCR, basal ECAR, intracellular ATP, extracellular lactate, and gene expression were assessed using one-way ANOVA, followed by Tukey’s family-wise-adjusted multiple comparisons test. Statistical comparisons correspond to the brackets shown or to the prespecified comparisons reported in the text. Figure 3 is treated as a six-group mechanistic comparison; no AZA × dnHIF interaction or synergy inference is made from this figure. ** *p* < 0.01, *** *p* < 0.001. FCCP, carbonyl cyanide-p-trifluoromethoxyphenylhydrazone; HRE, hypoxia-response element; OCR, oxygen consumption rate; ECAR, extracellular acidification rate.

To determine how tumor cells preserve bioenergetic capacity during AZA-associated respiratory stress, we first examined activation of the HIF transcriptional program. AZA increased HRE-luciferase activity from 0.98-fold in vehicle-treated cells to 2.17-fold, and this response was retained in cells receiving AZA together with G-LNP-delivered control mRNA (AZA+mCtrl; 2.08-fold). In contrast, cells treated with AZA and G-LNP-delivered dnHIF-1α mRNA (AZA+dnHIF) remained near baseline (0.97-fold versus 2.08-fold in AZA+mCtrl; Tukey-adjusted *p* < 0.0001; Figure 3A), confirming that dnHIF prevented AZA-induced HIF transcriptional activation.

We next determined whether HIF blockade altered the primary respiratory response to AZA. Seahorse analysis showed that AZA markedly suppressed mitochondrial respiration irrespective of HIF inhibition. Basal OCR decreased from 272.7 pmol min^−1^ in mCtrl cells to 159.8 pmol min^−1^ in AZA+mCtrl cells (Tukey-adjusted *p* < 0.0001), whereas AZA+dnHIF cells exhibited a similarly reduced basal OCR of 149.7 pmol min^−1^ (*p* = 0.98 versus AZA+mCtrl; Figure 3B). Thus, dnHIF did not measurably intensify the AZA-induced respiratory defect. Instead, its major metabolic effect was evident in the glycolytic response. Basal ECAR increased from 40.8 mpH min^−1^ in mCtrl cells to 75.1 mpH min^−1^ in AZA+mCtrl cells, consistent with compensatory glycolytic activation following mitochondrial inhibition. This response was abolished in AZA+dnHIF cells, in which basal ECAR decreased to 24.7 mpH min^−1^ (Tukey-adjusted *p* < 0.0001 versus AZA+mCtrl; Figure 3C). These findings distinguish the primary respiratory defect from the adaptive response: AZA suppresses mitochondrial respiration, whereas HIF activity is required for the accompanying increase in glycolytic activity.

Failure to mount this compensatory response substantially deepened the energetic deficit. Intracellular ATP was reduced to 77.7% of the vehicle level in AZA+mCtrl cells, but fell further to 50.1% in AZA+dnHIF cells (Tukey-adjusted *p* < 0.0001; Figure 3D). Extracellular lactate showed the reciprocal pattern, increasing to 23.4 nmol per 10^4^ viable cells in AZA+mCtrl cells, but remaining at 9.1 nmol per 10^4^ viable cells in AZA+dnHIF cells (*p* < 0.0001; Figure 3E). These findings indicate that the glycolytic response elicited by respiratory inhibition partially buffers ATP loss, rather than restoring mitochondrial function.

Consistent with this interpretation, AZA+mCtrl increased GLUT1, HK2, LDHA, and PDK1 expression levels to 2.12-, 1.81-, 1.58-, and 2.08-fold, respectively, whereas expression levels in AZA+dnHIF cells remained at or below baseline (0.85-, 0.81-, 0.82-, and 0.80-fold; all prespecified Tukey-adjusted comparisons *p* ≤ 0.0022; Figure 3F). The concordant suppression of HIF transcriptional activity, glycolytic gene induction, extracellular acidification, and lactate production identifies HIF-dependent glycolysis as an adaptive program engaged during AZA-induced respiratory stress. By disabling this program without materially increasing the primary respiratory defect, dnHIF removes a major bioenergetic buffer and converts partial ATP depletion into a substantially deeper energy deficit.

### 2.4. HIF Blockade Under AZA Stress Is Associated with a Distinct Metabolomic State

**Figure 4:**
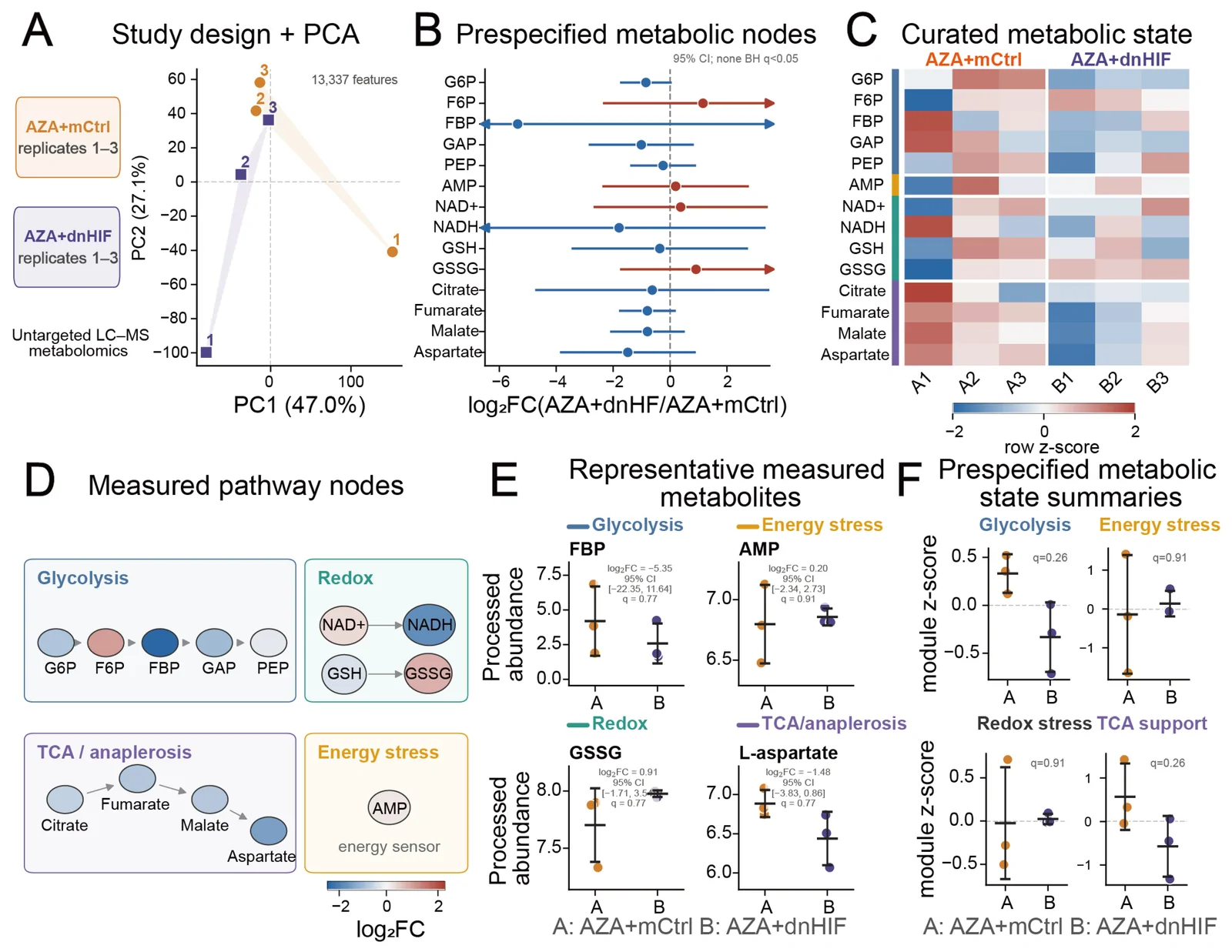
HIF blockade under AZA stress is associated with a distinct metabolomic state. (**A**) Study design and principal component analysis (PCA) of all 13,337 untargeted LC–MS features passing pooled QC RSD ≤ 30%, completeness and non-zero-variance filters. Each symbol represents one sample (*n* = 3 samples per group); features were centered and scaled before PCA and percentages indicate variance explained. (**B**) Log2 fold changes for 14 prespecified, confidently annotated metabolic nodes comparing AZA+dnHIF with AZA+mCtrl. Points show group-mean differences, and lines show two-sided Welch 95% confidence intervals; arrowheads indicate confidence intervals extending beyond the plotted range. Blue denotes lower and red denotes higher abundance in AZA+dnHIF. No displayed node passed BH *q* < 0.05 after correction across all 13,337 features. (**C**) Row-standardized abundance heatmap for the same 14 metabolites across all six samples. (**D**) Pathway-centered view of the measured annotated nodes. Node color encodes log_2_ fold change; equal node sizes are used throughout, and arrows indicate biochemical adjacency only, rather than measured flux. (**E**) Replicate-level supplier-processed abundance for four prespecified representative metabolites: fructose-1,6-bisphosphate, AMP, GSSG and L-aspartate. Dots represent biological samples; bars and whiskers indicate mean ± SD; and each panel reports log_2_ fold change, 95% confidence interval and BH q value. None passed feature-wide BH correction. (**F**) Prespecified metabolic state summaries calculated from explicitly defined z-scored metabolite sets. Dots represent individual samples, and bars/whiskers indicate mean ± SD; q values are BH-adjusted across the four summary comparisons. A, AZA+mCtrl; B, AZA+dnHIF. Exploratory pathway-level permutation ranking is shown in Supplementary Figure S3; no tested pathway reached BH *q* < 0.05.

To determine whether HIF blockade altered the metabolic response to AZA, we analyzed the six samples whose treatment identities were independently confirmed, comprising three AZA+mCtrl and three AZA+dnHIF samples. PCA of 13,337 features that passed the pooled QC variability threshold separated the two treatment groups along the principal axes, with PC1 and PC2 explaining 47.0% and 27.1% of the variance, respectively (Figure 4A). This global pattern indicated a treatment-associated shift in the measured metabolomic state, while the small group size warranted feature- and pathway-level multiple testing control.

Across the full feature set, 508 features had nominal *p* < 0.05, but only two remained significant after Benjamini–Hochberg correction; neither could be assigned a reliable metabolite identity from the supplied annotation table. We therefore focused the mechanistic display on 14 metabolites on the basis of annotation quality; pooled QC performance; and relevance to glycolysis, energetic stress, redox balance and TCA/anaplerotic metabolism. Eleven of these 14 nodes changed in the prespecified direction (Figure 4B,C). The strongest coherent pattern was lower in abundance across several glycolytic and TCA/anaplerotic intermediates in the AZA+dnHIF group, whereas AMP and GSSG tended to increase (Figure 4D). However, none of the individual prespecified metabolites passed feature-wide BH correction, and the wide confidence intervals emphasize the uncertainty associated with *n* = 3 samples per group.

Representative replicate-level measurements further illustrated the directional pattern observed in the curated metabolic map. Fructose-1,6-bisphosphate was lower in the AZA+dnHIF group, whereas AMP showed a modest increase. GSSG changed in the direction expected for increased oxidative stress, and L-aspartate was lower in the AZA+dnHIF group (Figure 4E). These individual metabolite measurements are presented descriptively because none passed feature-wide BH correction. Prespecified metabolic state summaries likewise showed lower glycolysis support and TCA/anaplerotic support means in AZA+dnHIF cells, whereas the energy and redox stress summaries changed only modestly; none of the four comparisons passed BH correction (Figure 4F).

Exploratory pathway-level permutation analysis did not identify any pathway that remained significant after BH correction (Supplementary Figure S3). Together, these data support an exploratory model in which HIF blockade under AZA stress is associated with a distinct central carbon metabolic state. Targeted quantitative metabolomics and independent biological replication will be required to confirm the individual metabolites, pathway assignments and functional flux consequences.

### 2.5. Failure of HIF-Dependent Adaptation Converts Transient Metabolic Stress into Durable Loss of Tumor Cell Fitness

**Figure 5:**
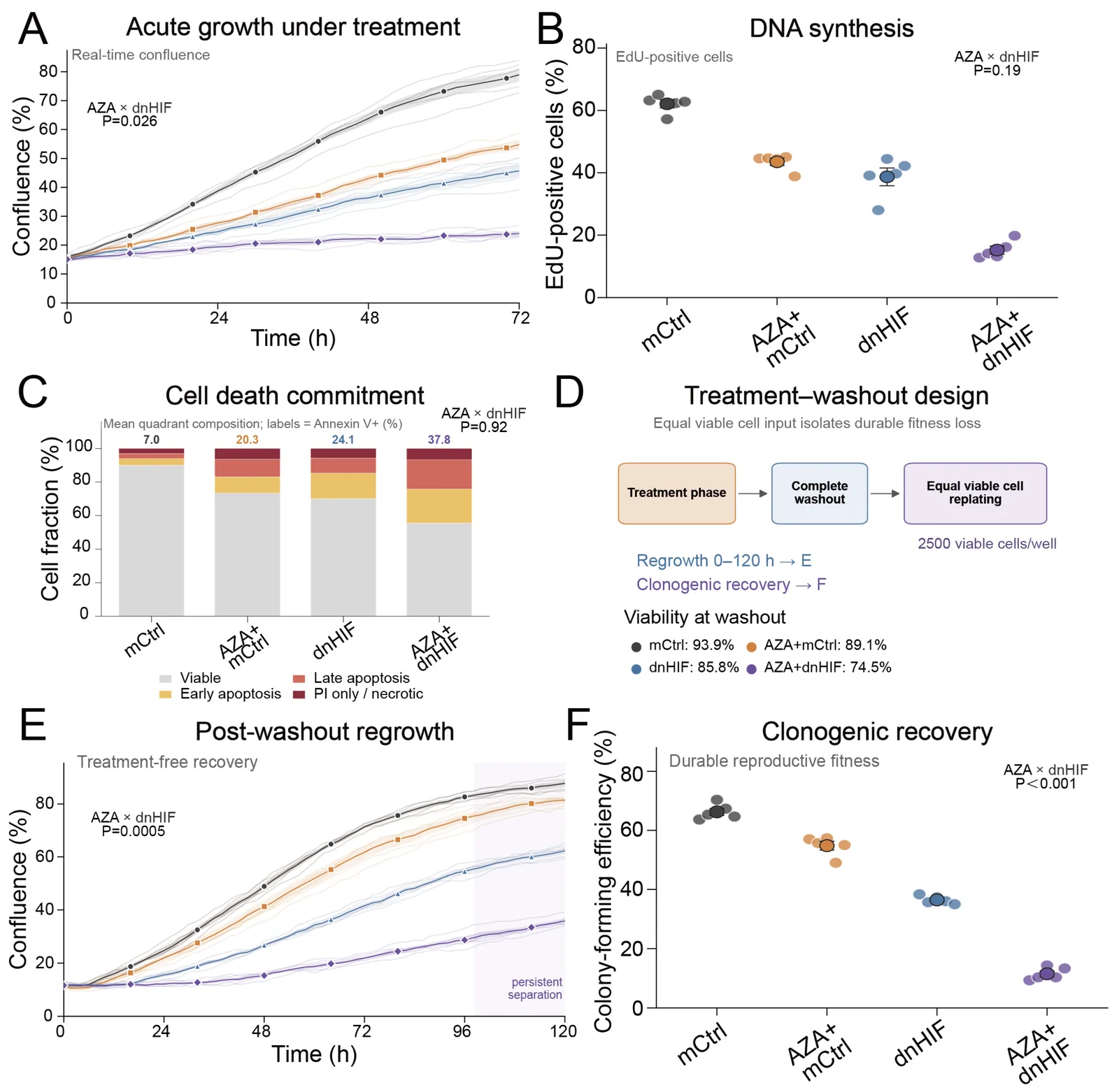
Failure of HIF-dependent adaptation converts transient metabolic stress into durable loss of tumor cell fitness. Cells were pre-exposed to mRNA-LNPs at an mRNA-equivalent concentration of 0.5 μg mL^−1^ for 12 h before AZA was added to a final concentration of 5 mM; the subsequent 72 h interval after AZA addition was defined as the acute treatment phase. Treatment-group colors are dark gray (mCtrl), orange (AZA+mCtrl), blue (dnHIF), and purple (AZA+dnHIF). (**A**) Real-time tumor cell confluence during treatment in the mCtrl, AZA+mCtrl, dnHIF, and AZA+dnHIF groups. Thick lines indicate group means, shaded regions indicate s.e.m., and faint lines represent independent biological replicates (*n* = 5 per group). The AZA × dnHIF interaction was tested using AUC, calculated independently for each biological replicate (*p* = 0.026). (**B**) Percentage of EdU-positive cells after treatment. Counts from five imaging fields were pooled within each biological replicate before statistical analysis. Dots represent independent biological replicates (*n* = 5 per group); center symbols and whiskers indicate mean ± s.e.m. The AZA × dnHIF interaction was not significant (*p* = 0.190). (**C**) Mean Annexin V/PI composition showing viable, early apoptotic, late apoptotic, and PI-only/necrotic fractions across independent biological replicates (*n* = 5 per group). In the stacked bars, light gray, yellow, salmon red, and dark red indicate viable, early apoptotic, late apoptotic, and PI-only/necrotic cells, respectively. Values above the bars indicate mean total Annexin V-positive fractions. The reported AZA × dnHIF interaction refers to total Annexin V positivity and was not significant (*p* = 0.920). (**D**) Treatment–washout workflow used to distinguish differences in acute viable cell yield from persistent changes in post-treatment proliferative fitness. Following complete treatment withdrawal, equal numbers of viable cells were replated for treatment-free regrowth (2500 viable cells per well) or clonogenic analysis (300 viable cells per dish). Values indicate mean viability at washout; the AZA × dnHIF interaction was significant (*p* = 0.00021). (**E**) Treatment-free regrowth for 120 h after washout and equal viable cell replating. Thick lines indicate group means, shaded regions indicate s.e.m., and faint lines represent independent biological replicates (*n* = 5 per group). The interaction was tested using recovery AUC calculated independently for each biological replicate (*p* = 0.00048). The pale purple background highlights the persistent separation between treatment groups during the late recovery period. (**F**) Colony-forming efficiency after treatment withdrawal and equal viable cell replating. Dots represent independent biological replicates (*n* = 5 per group); center symbols and whiskers indicate mean ± s.e.m. The AZA × dnHIF interaction was significant (*p* < 0.0001). Two-sided *p* values were obtained using ordinary least-squares two-factor models containing the main effects of AZA and dnHIF and their interaction. AUC, area under the curve; EdU, 5-ethynyl-2′-deoxyuridine; PI, propidium iodide.

Having observed a distinct treatment-associated metabolomic state after HIF blockade under AZA stress, we next asked whether this metabolic state affected only the acute treatment response or resulted in a persistent loss of proliferative fitness. Real-time imaging during treatment showed progressive separation among the four experimental conditions, with mean growth AUCs of 3600.1, 2533.1, 2199.1, and 1465.9 confluence% × h for mCtrl, AZA+mCtrl, dnHIF, and AZA+dnHIF cells, respectively. Factorial analysis identified a significant AZA × dnHIF interaction (*p* = 0.026; Figure 5A), providing statistical evidence that the effects of the two perturbations on cumulative cell expansion were not strictly additive.

Consistent with the growth phenotype, EdU incorporation decreased from 62.1% in mCtrl cells to 43.5% with AZA+mCtrl, 38.7% with dnHIF, and 15.2% with AZA+dnHIF (Figure 5B). The AZA × dnHIF interaction for EdU incorporation was not significant (*p* = 0.190), indicating marked suppression of DNA synthesis in the combined condition without statistical evidence of a non-additive interaction at this endpoint. Total Annexin V positivity increased in parallel, from 7.0% in mCtrl cells to 20.3%, 24.1%, and 37.8% in the AZA+mCtrl, dnHIF, and AZA+dnHIF groups, respectively (Figure 5C). The interaction was likewise not significant for Annexin V positivity (*p* = 0.920), consistent with an approximately additive increase during acute treatment. Thus, the reduced cumulative expansion observed with the combination was accompanied by both decreased proliferative activity and increased cell death-associated changes, although neither endpoint individually showed evidence of a non-additive interaction.

We next distinguished an acute reduction in viable cell yield from a persistent defect in the proliferative capacity of surviving cells. After complete treatment withdrawal, viable cells were quantified and equal numbers were replated under treatment-free conditions (Figure 5D). Mean viability at washout was 93.9% for mCtrl, 89.1% for AZA+mCtrl, 85.8% for dnHIF, and 74.5% for AZA+dnHIF cells, with a significant AZA × dnHIF interaction (*p* = 0.00021). Replating 2500 viable cells per well normalized the initial viable cell input and therefore allowed subsequent regrowth to be evaluated independently of differences in cell yield at the end of treatment.

Following treatment withdrawal, AZA+mCtrl cells retained substantial regrowth capacity, whereas dnHIF-treated cells recovered more slowly, and cells previously exposed to AZA+dnHIF remained persistently impaired throughout the 120 h observation period (Figure 5E). Mean recovery AUCs were 6643.3, 5874.3, 4182.5, and 2442.9 confluence% × h for mCtrl, AZA+mCtrl, dnHIF, and AZA+dnHIF cells, respectively. A significant AZA × dnHIF interaction was detected for recovery AUC (*p* = 0.00048), demonstrating that the post-treatment effects of the two perturbations were not strictly additive. The persistence of impaired regrowth after withdrawal of both interventions indicates that the combined treatment reduced post-treatment proliferative competence, rather than merely delaying expansion during treatment exposure.

Clonogenic analysis provided an independent long-term measure of this fitness defect. Colony-forming efficiency decreased from 66.3% in mCtrl cells to 54.8% with AZA+mCtrl and 36.5% with dnHIF, but fell to 11.5% following AZA+dnHIF treatment (AZA × dnHIF interaction, *p* < 0.0001; Figure 5F). Thus, whereas AZA-induced mitochondrial stress alone remained substantially recoverable after treatment withdrawal, disruption of HIF-dependent adaptation during AZA exposure produced a durable reduction in the proliferative capacity of surviving tumor cells. Together, these findings indicate that HIF-dependent metabolic adaptation influences not only the acute response to mitochondrial stress, but also the capacity of tumor cells to re-establish growth after that stress is removed.

### 2.6. Combined AZA and G-LNP@dnHIF-1α mRNA Enhances MC38 Tumor Control with Preliminary Systemic Tolerability

**Figure 6:**
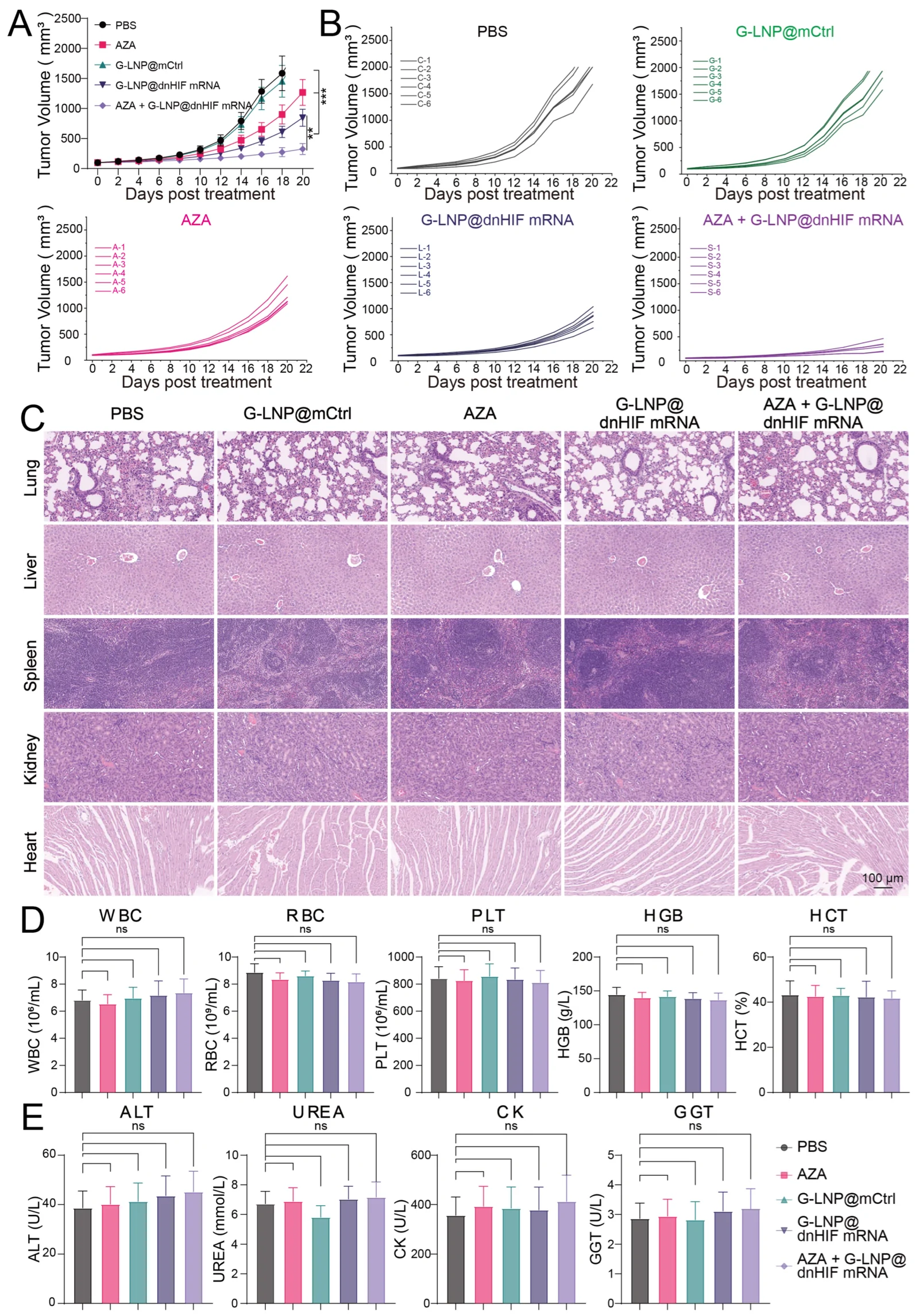
Combined AZA and G-LNP-delivered dnHIF-1α mRNA enhances MC38 tumor control with preliminary systemic tolerability. Mice bearing established subcutaneous MC38 tumors were treated with PBS, AZA, G-LNP@mCtrl, G-LNP@dnHIF-1α mRNA, or AZA plus G-LNP@dnHIF-1α mRNA (*n* = 6 mice per group). AZA was administered intraperitoneally, whereas G-LNP@mCtrl and G-LNP@dnHIF-1α mRNA were administered intratumorally. (**A**) Mean tumor volume trajectories during 20 days of follow-up. Data are presented as mean ± SD. Statistical comparisons at day 20 were performed using one-way ANOVA followed by Tukey’s multiple comparisons test; comparisons correspond to the brackets shown. (**B**) Individual tumor volume trajectories for all six mice in each treatment group. (**C**) Representative hematoxylin and eosin-stained sections of lung, liver, spleen, kidney, and heart collected at the study endpoint. Scale bar, 100 μm. (**D**) Complete blood count measurements, including white blood cell count (WBC), red blood cell count (RBC), platelet count (PLT), hemoglobin (HGB), and hematocrit (HCT) at the study endpoint (*n* = 6 mice per group). (**E**) Serum alanine aminotransferase (ALT), urea, creatine kinase (CK), and γ-glutamyltransferase (GGT) measured at the study endpoint (*n* = 6 mice per group). Body weight trajectories are presented in Supplementary Figure S4. Data in (**D**,**E**) are presented as mean ± SD. Statistical significance was assessed using one-way ANOVA, followed by Tukey’s multiple comparisons test; comparisons correspond to the brackets shown. Because the in vivo design did not include an AZA+G-LNP@mCtrl arm, it was not a complete 2 × 2 factorial experiment and no AZA × dnHIF interaction was inferred from this figure. ** *p* < 0.01, *** *p* < 0.001; ns, not significant. AZA, azelaic acid; ALT, alanine aminotransferase; CK, creatine kinase; GGT, γ-glutamyltransferase; HCT, hematocrit; HGB, hemoglobin; mCtrl, control mRNA; PLT, platelet count; RBC, red blood cell count; WBC, white blood cell count.

We next determined whether disruption of HIF-dependent metabolic adaptation could enhance the antitumor effect of AZA in vivo. Mice bearing established subcutaneous MC38 tumors were assigned to PBS, AZA, G-LNP@mCtrl, G-LNP@dnHIF-1α mRNA, or AZA plus G-LNP@dnHIF-1α mRNA groups (*n* = 6 per group) and followed for 20 days after treatment initiation (Figure 6A,B). AZA was administered intraperitoneally, whereas G-LNP@mCtrl and G-LNP@dnHIF-1α mRNA were administered intratumorally; the corresponding dosing schedules are described in Supporting Information, Section S19.

Tumors in the PBS and G-LNP@mCtrl groups grew rapidly and followed similar trajectories throughout the 20-day observation period (Figure 6A), indicating that the control LNP/mRNA formulation did not materially affect tumor growth under the tested conditions. On day 20, AZA monotherapy reduced mean tumor volume to approximately 1250 mm^3^, whereas G-LNP@dnHIF-1α mRNA produced a greater numerical reduction to approximately 850 mm^3^. The combined treatment produced the greatest tumor growth inhibition, with mean tumor volume remaining near 350 mm^3^ on day 20, significantly below that observed with either active monotherapy. These findings indicate that AZA-induced metabolic stress and blockade of HIF-dependent adaptation together provide greater tumor control than either intervention alone.

Individual tumor growth trajectories were consistent with the group-level response (Figure 6B). All tumors in the PBS and G-LNP@mCtrl groups showed rapid expansion during follow-up, whereas AZA and G-LNP@dnHIF-1α mRNA produced intermediate and more variable growth suppression. By contrast, tumors in the combination group remained consistently smaller throughout the later phase of observation and were below the corresponding monotherapy ranges at the end of follow-up. Thus, the enhanced group-level response was not attributable to a small subset of animals.

We next assessed systemic tolerability using organ histology, hematologic indices, serum biochemistry, and body weight monitoring. Hematoxylin and eosin staining of the lung, liver, spleen, kidney, and heart revealed no readily apparent treatment-associated inflammatory, necrotic, or degenerative abnormalities in the representative sections examined (Figure 6C). Consistent with these observations, white blood cell, red blood cell, and platelet counts; hemoglobin concentration; and hematocrit showed no significant differences among treatment groups (Figure 6D). Serum alanine aminotransferase, urea, creatine kinase, and γ-glutamyl transferase values were likewise comparable across groups (Figure 6E). Body weight trajectories were monitored in parallel and are presented in Supplementary Figure S4. Collectively, these measurements provide no evidence of overt systemic toxicity over the 20-day treatment period, while supporting preliminary tolerability of the tested regimen.

Taken together, the mean and individual tumor growth data show that combined AZA and G-LNP-delivered dnHIF-1α mRNA provides greater tumor control than either active treatment alone, while the available histologic, hematologic, biochemical, and body weight assessments support short-term systemic tolerability. These findings extend the cellular evidence of failed HIF-dependent metabolic adaptation and persistent fitness loss to an in vivo setting. However, the present study does not directly establish tumor-localized nanoparticle delivery, intratumoral dnHIF-1α expression, or in vivo HIF target engagement, and these remain important requirements for mechanistic validation of the therapeutic response.

## 3. Conclusions and Translational Outlook

This study advances our previously reported nano-energy interference (NEI) concept from downstream restriction of compensatory glucose utilization to delivery-enabled control of an upstream metabolic adaptation program [10]. The earlier DAF@LNP platform combined AZA-associated respiratory/metabolic stress with 2-DG-mediated restriction of glucose utilization and demonstrated that simultaneous interference with mitochondrial and glycolytic energy metabolism could suppress CRC growth and experimental liver metastasis. Here, we identify HIF-1α-dependent glycolytic compensation as a key adaptive response to AZA-associated respiratory impairment and use G-LNP-mediated dnHIF-1α mRNA delivery to transiently disrupt this response. Glutamine functionalization enhanced nanoparticle uptake and productive mRNA expression in ASCT2-expressing MC38 cells, while delivered dnHIF-1α prevented AZA-associated HIF transcriptional activation, glycolytic gene induction, extracellular acidification, and lactate production without materially intensifying the measured respiratory defect. Blocking this compensatory program deepened ATP depletion, was associated with a directionally coherent but exploratory change in central carbon metabolite profiles, and, importantly, impaired post-treatment regrowth and clonogenic capacity even after treatment withdrawal and equal viable cell replating. The same combination produced the greatest tumor growth inhibition among the tested regimens in subcutaneous MC38 tumors, with no overt abnormalities detected by the short-term systemic tolerability assessments performed.

These findings support a dynamic model of metabolic treatment response, in which therapeutic vulnerability is determined not only by the pathway initially perturbed, but also by the capacity of tumor cells to activate an alternative energy-producing program. The separation between the OCR and ECAR responses is particularly informative: dnHIF-1α did not substantially increase the respiratory impairment associated with AZA, but instead prevented the glycolytic adaptation that partially buffered ATP loss. The resulting phenotype should therefore be interpreted as failure of metabolic compensation, rather than simply greater respiratory inhibition. Untargeted metabolomic profiling further associated this state with a distinct, directionally coherent central carbon pattern, including lower glycolysis and TCA/anaplerosis support summaries; the named metabolites and prespecified module comparisons did not remain significant after BH correction and are therefore interpreted as exploratory. Because mitochondrial respiration also supports redox balance and biosynthetic functions beyond ATP generation [21], however, the relative contributions of ATP depletion, altered redox homeostasis, and biosynthetic insufficiency to the persistent fitness defect remain to be resolved experimentally.

An additional pharmacological limitation concerns the nominal 5 mM AZA exposure used in the in vitro mechanistic experiments. This concentration was used as an experimental respiratory/metabolic stress condition and should not be interpreted as pharmacokinetically equivalent to the 20 mg kg^−1^ intraperitoneal regimen used in vivo. AZA was prepared as a neutralized aqueous sodium azelate solution by addition of equimolar NaOH and adjustment to pH 7.4. However, sodium/osmolality-matched vehicle controls were not included, and contributions from ionic or osmotic conditions therefore cannot be completely excluded. Accordingly, the present study does not assign the phenotype to a specific mitochondrial molecular target. In a matched AZA concentration–response series, sodium/osmolality controls and comparison with an equi-effective canonical respiratory chain inhibitor will be required to establish pharmacological specificity and generalizability.

The delivery data also define important mechanistic and translational boundaries. The present results support enhanced, GPNA-sensitive and ASCT2-associated uptake of glutamine-functionalized LNPs, but do not establish ASCT2-specific receptor-mediated internalization. GPNA is not selective for ASCT2 [20], compensatory glutamine transport can occur after SLC1A5 loss [22], and the elevator-type transport mechanism of ASCT2 does not itself imply translocation of intact nanoparticles [23]. Genetic SLC1A5 loss-and-rescue experiments, ligand density studies, and direct binding and internalization measurements will therefore be required to define the contribution of ASCT2 to G-LNP uptake more rigorously. Likewise, the current in vivo study demonstrates therapeutic activity, but does not directly establish tumor-localized nanoparticle delivery, intratumoral dnHIF-1α expression, or HIF target engagement. Such validation is important because nanoparticle performance in vitro may correlate only weakly with in vivo delivery [24], and modest changes in LNP composition can substantially alter tissue tropism [15,16]. Entry into solid tumors remains a barrier to nanoparticle delivery [25], while productive cytosolic mRNA release requires overcoming intracellular delivery barriers [13,14]. Pharmacokinetic and biodistribution studies, cell type-resolved delivery analysis, and direct comparison with non-targeted LNPs in vivo will be necessary to determine whether the uptake advantage observed in culture translates into improved tumor-selective exposure.

In the context of existing mRNA nanomedicine platforms, the present formulation occupies an intermediate design space. Non-targeted ionizable LNPs provide a mature route to cytosolic mRNA expression, but generally lack an added tumor cell-recognition ligand and rely on inherent physicochemical and endogenous corona-mediated biodistribution. SORT nanoparticles can redirect delivery at the organ level [15,16], whereas ligand-decorated carriers attempt to improve cell-level association [19,26]; these strategies are complementary, rather than interchangeable. For example, peptide-functionalized LNPs have been used to deliver SIRPα-CAR mRNA intratumorally for in situ macrophage reprogramming [26]. The glutamine-functionalized polymer reported by Honda et al. established the feasibility of multivalent ASCT2-associated tumor interaction, but used a polymeric carrier and did not test therapeutic mRNA delivery [19]. G-LNPs combine a conventional ionizable LNP core, a low-density nutrient ligand, and a dominant-negative transcriptional regulator mRNA. This combination is conceptually distinct, but the present study does not yet provide a head-to-head in vivo comparison with non-targeted LNPs, SORT formulations, or antibody/peptide-targeted mRNA carriers. Such comparisons will be necessary before assigning a relative translational advantage.

A focused validation sequence would address the main remaining uncertainties. First, SLC1A5 knockout and rescue should be combined with fluorescent lipid and RNA label measurements, acid-wash or protease-stripping controls, and endosomal colocalization to separate binding from internalization and cytosolic release. Second, a replicated AZA-by-mRNA dose matrix should be analyzed with Bliss, Loewe, highest-single-agent, and ZIP models on both acute viability and post-washout clonogenic endpoints. Third, in vivo biodistribution should be measured using labeled LNPs or reporter mRNA with flow cytometric resolution of tumor, stromal, and immune compartments, together with intratumoral dnHIF-1α expression and HIF-target engagement. Finally, orthotopic and metastatic CRC models, human CRC organoids, and repeat-dose immunotoxicology studies will be needed to assess generalizability and safety.

Several additional issues will determine the translational scope of this strategy. Because both glutamine transport and HIF-1α-dependent metabolism contribute to immune cell function [27,28], the effects of G-LNP@dnHIF-1α mRNA on tumor-infiltrating immune and stromal populations should be resolved in immune-competent models. The absence of overt abnormalities in body weight, major organ histology, hematologic indices, and serum biochemistry over the present treatment period supports preliminary tolerability, but not comprehensive safety; repeat-dose studies should therefore assess inflammatory responses, complement activation, anti-PEG immunity, tissue accumulation, and recovery after treatment cessation. These studies should also account for the fact that nucleoside modification can reduce innate RNA sensing [29], whereas inflammatory activity can arise from the LNP formulation itself and varies with composition, dose, and route [30]. Therapeutic generalizability should also be tested in orthotopic and metastatic CRC models and across human CRC systems, including patient-derived organoids and xenografts. Prospective CRC data support the use of patient-derived organoids as a translational bridge because ex vivo responses can correlate with clinical treatment response [31]. Such studies will help determine whether ASCT2 abundance, basal HIF activity, mitochondrial dependence, or a composite metabolic profile can identify tumors most likely to respond to adaptive HIF blockade.

In conclusion, the present work extends NEI from downstream metabolic co-interference to programmable disruption of the adaptive program elicited by an experimentally imposed respiratory/metabolic stress. By distinguishing AZA-associated respiratory impairment from the HIF-dependent glycolytic response that buffers it, this study identifies metabolic adaptability itself as a therapeutically actionable vulnerability. Transient dnHIF-1α mRNA delivery converts otherwise partially recoverable AZA-associated respiratory/metabolic stress into persistent proliferative impairment associated with a distinct metabolomic state and enhanced tumor control. With direct in vivo target engagement evidence, rigorous validation of the delivery mechanism, and expanded pharmacologic and safety evaluation, this framework may provide a testable strategy for designing metabolic therapies that target not only an energy-producing pathway, but also the adaptive response that enables tumor cells to survive its disruption.

## Data Availability

The data that support the findings of this study are available from the corresponding authors upon reasonable request.

## Supplementary Materials

The following supporting information can be downloaded at: https://www.mdpi.com/article/10.3390/molecules31213791/s1, Figure S1: Flow cytometric analysis of cell-surface ASCT2 positivity in CMT-93, CT26, and MC38 cells; Figure S2: Glutamine functionalization enhances GFP mRNA transfection efficiency in MC38 cells; Figure S3. Exploratory pathway-level permutation ranking of the untargeted metabolomics comparison; Figure S4. Body-weight trajectories of MC38 tumor-bearing mice during treatment; Table S1: Primer sequences.
