# Supplementary material for: Glutamine-Functionalized mRNA Lipid Nanoparticles Block HIF-1α-Dependent Glycolytic Adaptation to Sensitize Azelaic Acid Therapy in Colorectal Cancer
Source: Molecules. 2026 Oct 22;31(21):3791. doi: 10.3390/molecules31213791 (PMC13764850; doi:10.3390/molecules31213791)
Supplement: Supplementary file 1 [file molecules-31-03791-s001.zip › molecules-4549263-supplementary.pdf]

Supporting Information for

Glutamine-functionalized mRNA lipid nanoparticles block HIF-1α-dependent glycolytic adaptation to sensitize azelaic acid therapy in colorectal cancer

Materials and Methods

S1. Ethics Statement

All animal experiments were conducted at Minhang Hospital, Fudan University. The experimental protocols involving animals were reviewed and approved by the Department of Laboratory Animal Science, Fudan University (approval No. 2023-MHFY-18JZS). All animal procedures were performed in accordance with the approved experimental protocol and the institutional regulations for the care and use of laboratory animals.

S2. Materials and key reagents

SM-102 (Cayman Chemical, Ann Arbor, MI, USA; item no. 33474), DSPC (Avanti Polar Lipids/Avanti Research; legacy product no. 850365/current code A80365), and cholesterol (Sigma-Aldrich; catalog no. C8667) were used for LNP preparation. mPEG-DMG, azelaic acid (AZA), L-γ-glutamyl-p-nitroanilide (GPNA), and additional analytical reagents were of analytical or molecular-biology grade as appropriate. Molecular-biology-grade water and endotoxin-free plasticware were used for mRNA-containing and in vivo formulations.

S3. Design and preparation of mRNA

The dominant-negative HIF-1α (dnHIF-1α) mRNA used in this study encoded a human HIF-1α-no-TAD construct designed according to the strategy reported by Brown et al. [1]. The construct retained amino acids 1–390 and lacked amino acids 391–826 of HIF-1α. The DNA sequence of the linker at the deletion junction was 5′-TTAAGTGAGCTTTTTCTTAATCTAGAGC-3′. GFP mRNA was used as a reporter to evaluate productive intracellular mRNA delivery, whereas a commercially synthesized

non-targeting control mRNA (mCtrl; Sangon Biotech, Shanghai, China) was used as the negative control in mechanistic experiments. All mRNAs used in this study were aliquoted under RNase-free conditions, stored at −80 °C, and thawed only as required before formulation preparation.

S4. Preparation of Gln-PEG-DMG-functionalized mRNA lipid nanoparticles

Gln-PEG-DMG was custom synthesized by Baotian Biotechnology (Shanghai) Co., Ltd. (Shanghai, China) and used without further chemical modification. SM-102, DSPC, cholesterol, Gln-PEG-DMG, and mPEG-DMG were dissolved in ethanol at a molar ratio of 50:10:38.5:0.3:1.2. mRNA was diluted in 25 mM sodium acetate buffer (pH 4.0), and the ionizable-lipid nitrogen-to-mRNA phosphate ratio (N/P ratio) was adjusted to 6. The aqueous and organic phases were combined by microfluidic mixing using an INano L^{TM} microfluidic system (Micro & Nano Instrument Technology Co., Ltd., Shanghai, China) at an aqueous-to-organic flow-rate ratio of 3:1 and a total flow rate of 12 mL min^{-1}.

The freshly prepared nanoparticle suspension was subsequently diluted with phosphate-buffered saline (PBS), concentrated by ultrafiltration, and buffer-exchanged into PBS. Sucrose was added to a final concentration of 8% (w/v). The resulting formulation was designated G-LNP@dnHIF-1α mRNA. GFP mRNA or control mRNA (mCtrl) was substituted for dnHIF-1α mRNA using the same formulation procedure where indicated.

Non-targeted LNPs were prepared using the same procedure except that Gln-PEG-DMG was omitted and replaced with an equimolar amount of mPEG-DMG. For fluorescence-tracking experiments, FITC-conjugated cholesterol was incorporated into the lipid phase by replacing an equivalent molar fraction of unlabeled cholesterol.

S5. Physicochemical characterization of LNPs

S5.1 Hydrodynamic diameter, polydispersity, and zeta potential

LNPs were diluted in sterile-filtered dispersant and equilibrated at 25 °C before dynamic light scattering (DLS). Hydrodynamic size and polydispersity were measured using a backscatter DLS configuration, and zeta potential was measured by laser Doppler electrophoresis. Technical acquisitions within a batch were averaged to obtain

one value for each independently prepared batch.

S5.2 Transmission electron microscopy

For transmission electron microscopy (TEM), 5 μL of diluted LNP suspension was applied to a glow-discharged carbon-coated copper grid for 1 min. Excess liquid was removed, and the grid was negatively stained with 1% (w/v) phosphotungstic acid (pH 7.0) for 30 s and air-dried. Particle diameter was quantified from non-overlapping micrographs using ImageJ/Fiji. A total of 343 particles were analyzed for the particle-size distribution shown in Figure 1C, D.

S5.3 mRNA encapsulation efficiency

mRNA encapsulation efficiency was quantified using the Quant-iT RiboGreen RNA Assay Kit (Invitrogen/Thermo Fisher Scientific, catalog no. R11490) using paired measurements of intact LNPs and detergent-disrupted LNPs. Samples were analyzed without detergent to quantify accessible/unencapsulated mRNA and after disruption with 0.5% Triton X-100 to quantify total mRNA. Encapsulation efficiency was calculated as 100 × [1 − (free mRNA/total mRNA)]. The tested initial mRNA concentrations were 0.05, 0.10, 0.20, 0.30, and 0.40 mg mL^{-1} while maintaining N/P = 6.

S5.4 Colloidal stability

For colloidal-stability measurements, LNPs were incubated in PBS or complete DMEM containing 10% fetal bovine serum, and hydrodynamic size was monitored for 14 days.

S6. Cell culture and treatment scheme

CMT-93, CT26.WT, and MC38 murine CRC cells were maintained at 37 °C in a humidified atmosphere containing 5% CO_2. CMT-93 and MC38 cells were cultured in DMEM, whereas CT26.WT cells were cultured in RPMI-1640, with both media supplemented with 10% heat-inactivated fetal bovine serum.

AZA was prepared as a neutralized aqueous sodium azelate solution by neutralization with NaOH, followed by adjustment to pH 7.4 and sterile filtration immediately before use. Unless otherwise specified, G-LNP@mCtrl and G-LNP@dnHIF-1α mRNA were applied at a final mRNA concentration of 0.5 μg mL^{-1}.

For the principal mechanistic experiments, MC38 cells were pre-exposed to the indicated mRNA-LNP formulation for 12 h before AZA was added to a final concentration of 5 mM. Acute mechanistic endpoints, including HIF-responsive reporter activity, extracellular flux, intracellular ATP, extracellular lactate, RT-qPCR, and untargeted LC-MS metabolomic profiling, were assessed 24 h after AZA addition. For the cell-growth, EdU-incorporation, Annexin V/PI, and treatment-washout experiments, AZA exposure was continued for 72 h, with time 0 defined as the time of AZA addition.

S7. Flow-cytometric analysis of cell-surface ASCT2

CMT-93, CT26.WT, and MC38 cells were detached under conditions that preserved cell-surface proteins, washed in ice-cold staining buffer, blocked with anti-mouse CD16/32, and stained at 4 °C with a fluorophore-conjugated anti-mouse SLC1A5/ASCT2 antibody together with a viability dye. An ASCT2 fluorescence-minus-one (FMO) control and a fluorophore- and concentration-matched isotype control were included. Singlets and viable cells were identified using a common gating template, and the ASCT2-positive threshold was defined using the FMO control and applied identically to all samples. ASCT2-positive fractions were quantified by flow cytometry.

S8. Cellular association and uptake of FITC-labeled LNPs

MC38 cells were seeded on imaging-compatible culture ware 24 h before nanoparticle exposure. FITC-labeled LNPs were applied at an mRNA-equivalent concentration of 0.5 µg mL^{-1}. For the time-course experiment, cells were incubated with FITC-labeled G-LNP@dnHIF-1α mRNA and imaged after 3, 6, 9, or 12 h. For competition experiments, cells were pretreated with 1 mM GPNA for 30 min before addition of G-LNPs, and GPNA was maintained at 1 mM throughout the subsequent 12 h nanoparticle exposure. Non-targeted FITC-LNPs were analyzed in parallel. After treatment, cells were washed, fixed in 4% paraformaldehyde, and, for high-magnification imaging, permeabilized, blocked, immunostained for α-tubulin, and counterstained with Hoechst 33342. Imaging settings were held constant across groups within an independent experiment. Background-corrected green mean fluorescence

intensity (MFI) and the percentage of FITC-positive cells were quantified using a fixed analysis pipeline and a positivity threshold defined from control samples.

S9. GFP reporter-mRNA expression

To assess productive cytosolic delivery and translation, MC38 cells were treated with non-targeted LNP@GFP mRNA or G-LNP@GFP mRNA at a matched final mRNA concentration of 0.5 μg mL^{-1}. For GPNA competition experiments, cells were pretreated with 1 mM GPNA for 30 min, and GPNA was maintained during the subsequent 12 h nanoparticle exposure. GFP fluorescence was imaged 12 h after nanoparticle addition for the targeting comparison. A separate 24 h formulation comparison included Lipofectamine 3000-complexed GFP mRNA, non-targeted LNP@GFP mRNA, and G-LNP@GFP mRNA at the same mRNA dose. GFP MFI was background-corrected, and the GFP-positive fraction was determined using a common threshold defined from untreated cells.

S10. HIF-responsive dual-luciferase reporter assay

MC38 cells were transiently cotransfected with an HRE-driven firefly-luciferase reporter and a constitutive Renilla-luciferase plasmid using Lipofectamine 3000. Following recovery from plasmid transfection, cells were pre-exposed to G-LNP@mCtrl or G-LNP@dnHIF-1α mRNA (0.5 μg mRNA mL^{-1}) for 12 h, followed by treatment with 5 mM AZA for 24 h. Firefly and Renilla activities were measured using a dual-luciferase assay, and firefly activity was normalized to Renilla activity for each well before expression relative to the vehicle group. Technical wells were averaged to generate one value per independent biological experiment (Figure 3A, $n = 6$).

S11. Extracellular flux analysis

Oxygen-consumption rate (OCR) and extracellular-acidification rate (ECAR) were measured using a Seahorse XF extracellular-flux analyzer. MC38 cells were seeded in Seahorse microplates and treated according to the principal treatment scheme described above (0.5 μg mRNA mL^{-1}, 12 h mRNA-LNP pre-exposure followed by addition of AZA to a final concentration of 5 mM and incubation for an additional 24 h). On the assay day, cells were washed and equilibrated for 60 min at 37 °C in a non-CO_2 incubator in bicarbonate-free XF DMEM (pH 7.4) containing 10 mM glucose, 2

mM glutamine, and 1 mM sodium pyruvate. Sensor cartridges were hydrated and calibrated according to the instrument protocol.

After baseline measurements, oligomycin, FCCP, and rotenone/antimycin A were sequentially injected according to the Seahorse Cell Mito Stress Test workflow. Final concentrations were 1.5 μM oligomycin, 1.0 μM FCCP, and 0.5 μM rotenone plus 0.5 μM antimycin A. ECAR was recorded concurrently with OCR and was interpreted as an index of glycolysis-associated extracellular acidification rather than as a dedicated measure of glycolytic capacity.

S12. Intracellular ATP and extracellular lactate

Intracellular ATP was quantified using a luminescence-based ATP assay (CellTiter-Glo 2.0, Promega, catalog no. G9241). Cells and reagent were equilibrated to room temperature, an equal volume of reagent was added to the culture medium, plates were mixed for 2 min and incubated for 10 min to stabilize the luminescent signal, and luminescence was recorded. Blank-corrected ATP values were expressed relative to the vehicle group (Figure 3D, $n = 6$).

For lactate measurements, conditioned medium collected over the 24 h treatment interval was clarified by low-speed centrifugation and analyzed using an L-lactate assay against a fresh standard curve. Medium-only blanks were subtracted, and lactate release was normalized to the viable-cell number in the corresponding well and reported as nmol per 10^4 viable cells over 24 h (Figure 3E, $n = 6$).

S13. RNA isolation and RT-qPCR

Total RNA was isolated from treated MC38 cells after 12 h of mRNA-LNP pre-exposure followed by 24 h of treatment with 5 mM AZA, using an RNase-free extraction procedure with DNase treatment. RNA quantity and purity were measured spectrophotometrically, and cDNA was generated from 1 μg total RNA. Quantitative PCR was performed with SYBR Green chemistry using gene-specific primers. No-template and no-reverse-transcriptase controls were included, and melt-curve analysis was used to verify a single predominant amplicon. Relative gene expression was calculated using the $2^{-\Delta\Delta Ct}$ method with *Rplp0* as the reference gene. Primer sequences are provided in Supplementary Table S1.

S14. Untargeted LC-MS metabolomic profiling and exploratory pathway analysis

Untargeted LC-MS profiling was performed on samples from the AZA+mCtrl and AZA+dnHIF groups, with three independent biological replicates per group. Sample identities were independently verified. Cells were collected 24 h after AZA addition following the 12 h mRNA-LNP pre-exposure described in the cell-treatment scheme. Untargeted data were acquired by the service provider using a Thermo Vanquish Horizon UHPLC coupled to a Q Exactive HF-X mass spectrometer in positive- and negative-ion electrospray-ionization modes. Peak detection and integration, retention-time correction, and feature alignment were performed in Progenesis QI v3.0.

The supplier-processed combined positive/negative-mode feature-abundance matrix was filtered using pooled-QC relative standard deviation (RSD) $\leq$ 30%, complete observations across all six samples, and non-zero variance. These filters retained 13,337 features for principal component analysis (PCA) and feature-level comparisons. The supplier documentation did not explicitly state the logarithm base of the processed matrix. We therefore compared 61,593 matched non-zero raw peak-area entries with their corresponding processed values. The processed values showed a Pearson correlation of 0.995 with $\log_{10}$(raw peak area) and a median offset of 0.109; the matrix was therefore treated as $\log_{10}$-like normalized abundance and was not subjected to a second logarithmic transformation. Group differences on this scale were converted to $\log_2$ fold changes as $\log_2FC$ = (mean $\log_{10}$ abundance in AZA+dnHIF − mean $\log_{10}$ abundance in AZA+mCtrl) × $\log_2(10)$.

S15. Metabolite annotation and pathway membership

Fourteen metabolites were selected based on annotation quality, pooled-QC performance, and predefined metabolic relevance. The set comprised glucose-6-phosphate (G6P), fructose-6-phosphate (F6P), fructose-1,6-bisphosphate (FBP), glyceraldehyde-3-phosphate (GAP), phosphoenolpyruvate (PEP), AMP, NAD^+, NADH, GSH, GSSG, citrate, fumarate, malate, and L-aspartate. Supplier-processed replicate-level values were retained for each of the six samples. Metabolite identities were taken from the supplier annotation table; pathway membership for exploratory ranking was

derived from the supplier's KEGG annotations.

S16. Prespecified metabolite comparisons and Figure 4F metabolic-state summaries

For the 14 prespecified metabolites, group differences between AZA+dnHIF and AZA+mCtrl were summarized using replicate-level supplier-processed abundances. Figure 4B reports group-mean differences as $\log_2$ fold changes with two-sided Welch 95% confidence intervals. Four representative metabolites (FBP, AMP, GSSG, and L-aspartate) are displayed in Figure 4E with all six biological samples, mean ± SD, $\log_2$ fold change, 95% confidence interval, raw *P* value, and Benjamini-Hochberg (BH)-adjusted *q* value. Across the complete 13,337-feature universe, two-sided Welch *t* tests were followed by BH correction. Of the 13,337 tested features, 508 had nominal $P < 0.05$ and two had BH $q < 0.05$; neither of the latter could be assigned a reliable metabolite identity from the supplied annotation table. None of the 14 prespecified metabolites passed feature-wide BH correction.

For Figure 4F, each metabolite was z-standardized across the six samples before summary scores were calculated, using z(x) = [x − mean(x across all six samples)]/SD(x across all six samples). The four descriptive summaries were calculated exactly as follows: Glycolysis support = mean[z(G6P), z(F6P), z(FBP), z(GAP), z(PEP)]; Energy stress = z(AMP); Redox stress = mean[−z(NAD^+), z(NADH), −z(GSH), z(GSSG)]; and TCA/anaplerotic support = mean[z(citrate), z(fumarate), z(malate), z(L-aspartate)]. The four summary comparisons were evaluated on individual-sample scores, and BH correction was applied across the four comparisons. These summaries are descriptive and are not presented as validated composite biomarkers; none of the four comparisons reached BH $q < 0.05$.

For exploratory pathway-level analysis, pathway membership was derived from the supplier's KEGG annotations. All possible 3-versus-3 sample-label allocations were evaluated for the 114 tested pathways, and the resulting pathway-level rankings were corrected by the BH procedure. No pathway remained significant after correction. This analysis is presented in Supplementary Figure S3 as exploratory ranking rather than as evidence of statistically established pathway enrichment.

S17. Acute growth, DNA synthesis, cell death, and post-treatment fitness

For all experiments in Figure 5, MC38 cells were pre-exposed to G-LNP@mCtrl or G-LNP@dnHIF-1α mRNA (0.5 μg mRNA mL^{-1}) for 12 h, after which 5 mM AZA or the corresponding vehicle was added. The subsequent 72 h treatment interval was defined as the acute-treatment phase, with time 0 corresponding to AZA addition.

S17.1. Real-time cell growth during treatment

MC38 cells were exposed to mCtrl, AZA+mCtrl, dnHIF, or AZA+dnHIF conditions and monitored by automated live-cell imaging for 72 h. Confluence was quantified using fixed segmentation parameters, and the area under the confluence-time curve (AUC) was calculated independently for each biological replicate using the trapezoidal rule (Figure 5A; $n = 5$ per group).

S17.2. EdU incorporation

DNA synthesis was measured by 5-ethynyl-2′-deoxyuridine (EdU) incorporation at the end of the 72 h treatment interval. Cells were pulsed with EdU, fixed, permeabilized, and subjected to click-chemistry detection according to the assay protocol. Nuclei were counterstained, and the EdU-positive fraction was determined from coded images using a common threshold. Counts from multiple fields within an independent experiment were pooled before calculating one biological-replicate value (Figure 5B; $n = 5$ per group).

S17.3. Annexin V/PI analysis

Cell-death-associated changes were assessed by dual Annexin V/propidium iodide (PI) staining at the end of the 72 h treatment interval. Floating and adherent cells were collected together, washed with cold PBS, resuspended in calcium-containing binding buffer, stained with Annexin V and PI in the dark, and analyzed by flow cytometry using single-stained controls for compensation and quadrant placement. Cells were classified as Annexin V^-/PI^-, Annexin V^+/PI^-, Annexin V^+/PI^+, or Annexin V^-/PI^+, corresponding operationally to viable, early-apoptotic, late-apoptotic, and PI-only/necrotic fractions. Total Annexin V positivity was defined as the sum of Annexin V^+/PI^- and Annexin V^+/PI^+ fractions (Figure 5C; $n = 5$ per group).

S17.4. Treatment washout, equal viable-cell replating, and regrowth

To distinguish acute treatment effects from persistent loss of proliferative competence, cells were subjected to complete treatment washout at the end of the designated treatment period. Cells were collected, and viability was determined before replating. Equal numbers of viable cells (2,500 cells per well) were replated under treatment-free conditions. Confluence was then monitored for 120 h by automated live-cell imaging using the same analysis settings across groups. Recovery AUC was calculated independently for each biological replicate (Figure 5D, E; $n = 5$ per group).

S17.5. Clonogenic recovery assay

For clonogenic recovery, equal numbers of viable cells (300 cells per dish) collected after completion of the 72 h treatment interval and complete treatment washout were replated in drug-free medium at low density and cultured until discrete colonies formed. Colonies were then fixed, stained, and counted. Colony-forming efficiency was calculated as 100 × (number of colonies/number of viable cells plated) for each independent biological replicate (Figure 5F; $n = 5$ per group).

S18. Animals and subcutaneous MC38 tumor model

Female C57BL/6J mice (6–8 weeks old) were purchased from Shanghai Lingchang Animal Co., Ltd. and used to establish a syngeneic subcutaneous MC38 tumor model. MC38 cells in logarithmic growth were harvested, washed, and injected subcutaneously into the flank. Tumor dimensions were measured with digital calipers, and tumor volume was calculated as (length × width^2)/2.

When tumors reached approximately 80–100 mm^3, mice were randomly assigned to five groups ($n = 6$ per group): PBS, AZA, G-LNP@mCtrl, G-LNP@dnHIF-1α mRNA, and AZA + G-LNP@dnHIF-1α mRNA.

S19. In vivo treatment and monitoring

AZA was administered intraperitoneally at 20 mg kg^{-1} once daily, and G-LNP@mCtrl or G-LNP@dnHIF-1α mRNA was administered intratumorally at 10 μg mRNA per tumor in 50 μL every 3 days. Combination-treated mice received both agents on their respective schedules, and PBS controls received volume-matched vehicle. Tumor size and body weight were monitored every 2 days, and general condition and

injection-site abnormalities were assessed throughout treatment. A tumor volume of ≥ 2000 mm^3 was the prespecified humane endpoint. Animals reaching this endpoint were euthanized immediately after the corresponding tumor-volume measurement by CO_2 inhalation. At the planned study endpoint, all remaining animals were euthanized by CO_2 inhalation immediately after the final measurements. Tumor-volume measurements and outcome assessments were not blinded to treatment allocation.

S20. Hematology, serum biochemistry, and histology

At the study endpoint, whole blood collected into EDTA tubes was analyzed for white blood cell count, red blood cell count, platelet count, hemoglobin concentration, and hematocrit. Serum was prepared from clotted blood and analyzed for alanine aminotransferase, urea, creatine kinase, and γ-glutamyltransferase.

Lung, liver, spleen, kidney, and heart were harvested at necropsy, fixed in 10% neutral-buffered formalin, processed routinely, embedded in paraffin, sectioned, and stained with hematoxylin and eosin. Representative fields were selected after review.

S21. Statistical analysis

Statistical analyses for non-omics datasets were performed using GraphPad Prism. Untargeted LC-MS analyses were performed using the filtering, feature-level testing, annotation, and summary-score workflow described above. Data are presented as mean ± SD unless otherwise specified in the figure legends; time-course data are presented as mean ± s.e.m. where indicated. Statistical tests were two-sided, and $P < 0.05$ was considered statistically significant. *$P < 0.05$, **$P < 0.01$ and ***$P < 0.001$; ns, not significant.

For Figure 2, uptake and reporter-expression comparisons were analyzed by one-way ANOVA followed by Tukey-adjusted multiple comparisons. For Figure 3, HRE activity, basal OCR, basal ECAR, intracellular ATP, extracellular lactate, and each RT-qPCR target were analyzed by one-way ANOVA followed by Tukey family-wise adjusted comparisons, using one value per independent biological experiment.

For Figure 4, PCA was calculated from the 13,337 filtered untargeted LC-MS features after centering and scaling. Feature-level differences between AZA+dnHIF and AZA+mCtrl were tested using two-sided Welch t tests followed by BH correction

across all 13,337 features. The 14 selected metabolites were used for descriptive display, with BH adjustment performed across all 13,337 tested features rather than separately for this subset. Figure 4F scores were calculated from the explicitly defined z-scored metabolite sets, and BH correction was applied across the four summary comparisons. Exploratory pathway ranking used supplier KEGG annotations and all possible 3-versus-3 sample-label allocations across 114 tested pathways; no pathway remained significant after BH correction.

For Figure 5, AUC or endpoint values from each independent biological replicate were analyzed using an ordinary least-squares two-factor model containing the main effects of AZA and dnHIF and the AZA × dnHIF interaction. The interaction term was interpreted as evidence for departure from additivity only when statistically significant. This model was applied to growth AUC, EdU-positive fraction, total Annexin V-positive fraction, viability at washout, recovery AUC, and colony-forming efficiency.

For Figure 6, prespecified day-20 tumor-volume comparisons and endpoint hematology/serum chemistry measurements were analyzed by one-way ANOVA followed by Tukey multiple-comparisons testing. Tumor-growth trajectories are shown for descriptive longitudinal visualization.

References

[1] Brown LM, Cowen RL, Debray C, et al. Reversing hypoxic cell chemoresistance in vitro using genetic and small molecule approaches targeting hypoxia inducible factor-1. Mol Pharmacol. 2006;69(2):411-418. doi:10.1124/mol.105.015743.

Supplementary Table S1. Primer sequences

Target	Gene	Forward primer (5′–3′)	Reverse primer (5′–3′)
GLUT1	*Slc2a1*	GCAGTTCGGCTATAACACTGG	GCGGTGGTTCCATGTTTGATTG
HK2	*Hk2*	TGATCGCCTGCTTATTCACGG	AACCGCCTAGAAATCTCCAGA
LDHA	*Ldha*	TGTCTCCAGCAAAGACTACTGT	GACTGTACTTGACAATGTTGGGA
PDK1	*Pdk1*	GGACTTCGGGTCAGTGAATGC	TCCTGAGAAGATTGTCGGGGA
Reference	*Rplp0*	AGATTCGGGATATGCTGTTGGC	TCGGGTCCTAGACCAGTGTTC

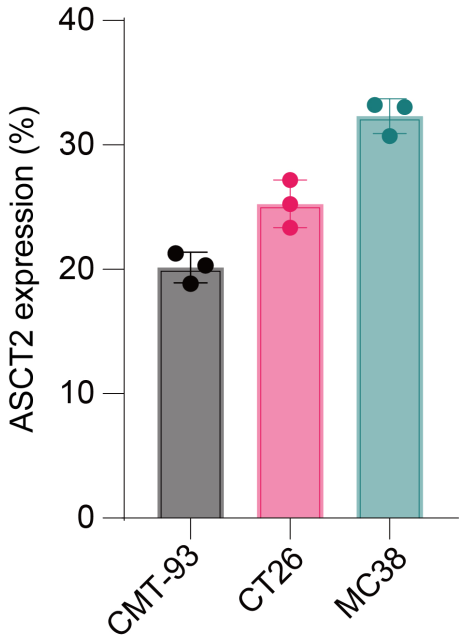

Supplementary Figure S1. Flow cytometric analysis of cell-surface ASCT2 positivity in CMT-93, CT26, and MC38 cells.

Bars represent the mean percentage of ASCT2-positive cells, error bars indicate SD, and overlaid dots represent independent biological experiments (*n* = 3).

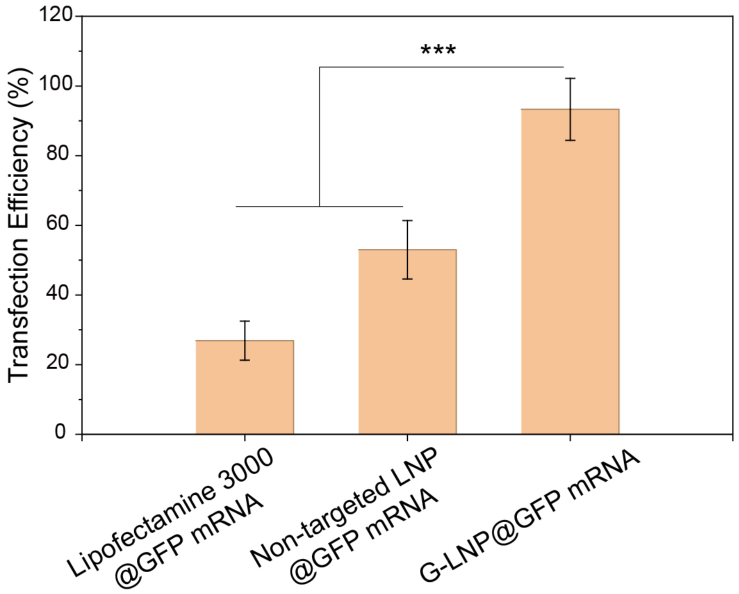

Supplementary Figure S2. Glutamine functionalization enhances GFP mRNA transfection efficiency in MC38 cells.

MC38 cells were treated with GFP mRNA delivered using Lipofectamine 3000, non-targeted LNP@GFP mRNA, or G-LNP@GFP mRNA and analyzed 24 h after transfection. Transfection efficiency was quantified as the percentage of GFP-positive cells. Data are presented as mean ± SD. ***$P < 0.001$ for the indicated comparison.

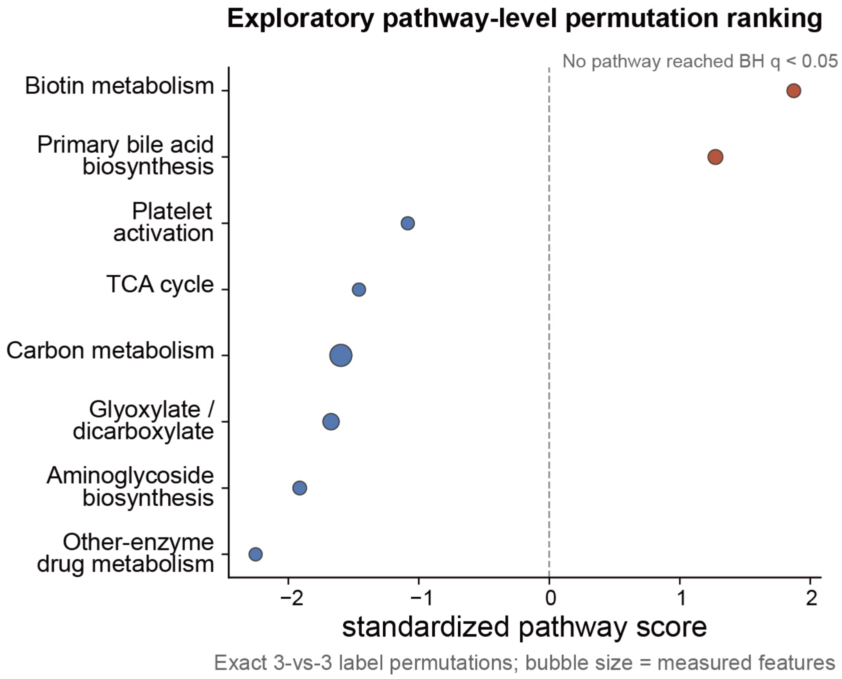

Supplementary Figure S3. Exploratory pathway-level permutation ranking of the untargeted metabolomics comparison.

Pathway membership was assigned using the supplier-provided KEGG annotations. Pathway-level scores were evaluated across all exact 3-versus-3 label permutations of the six samples, comprising three AZA+mCtrl samples and three AZA+dnHIF samples. The x-axis shows the standardized pathway score, and the dashed vertical line marks zero. Orange and blue bubbles indicate positive and negative standardized scores, respectively; bubble size is proportional to the number of measured LC–MS features contributing to each pathway. The displayed pathways are ordered by their standardized pathway scores and represent the ranked pathways selected for visualization from the 114 tested pathways. Benjamini–Hochberg correction was applied across all 114 pathway tests, and no pathway reached BH $q < 0.05$. This analysis is exploratory and is not interpreted as statistically established pathway enrichment.

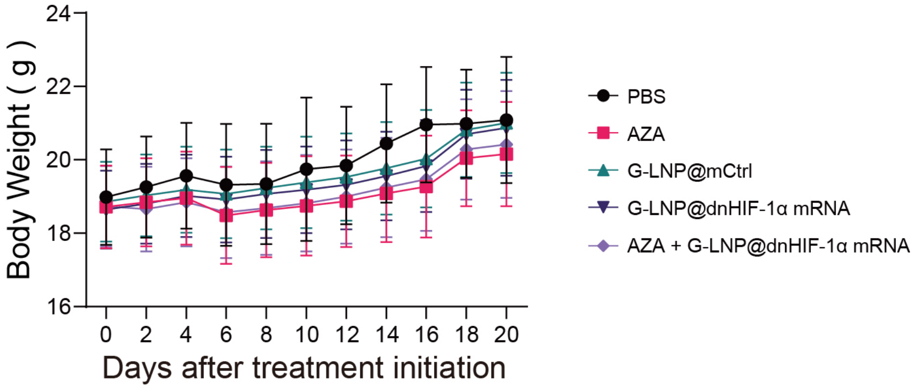

Supplementary Figure S4. Body-weight trajectories of MC38 tumor-bearing mice during treatment.

C57BL/6J mice bearing established subcutaneous MC38 tumors were treated with PBS, AZA, G-LNP@mCtrl, G-LNP@dnHIF-1α mRNA, or AZA plus G-LNP@dnHIF-1α mRNA (n = 6 mice per group). Body weight was recorded every 2 days throughout the 20-day treatment period. Data are presented as mean ± SD.
